# Supplementary material for: Single-cell transcriptomics uncovers a non-autonomous Tbx1-dependent genetic program controlling cardiac neural crest cell development
Source: Nat Commun. 2023 Mar 21;14:1551. doi: 10.1038/s41467-023-37015-9 (PMC10027855; doi:10.1038/s41467-023-37015-9)
Supplement: Supplementary file 1 — Supplementary Information [file 41467_2023_37015_MOESM1_ESM.pdf]

Supplementary Information

Single-cell transcriptomics uncovers a non-autonomous *Tbx1*-dependent genetic program controlling cardiac neural crest cell development

Christopher De Bono[1,*], Yang Liu[1], Alexander Ferrena[1,2], Aneesa Valentine[1], Deyou Zheng[1,3,4] and Bernice E. Morrow[1,5,*]

[1] Department of Genetics, Albert Einstein College of Medicine, Bronx, NY, USA.
[2] Institute for Clinical and Translational Research, Albert Einstein College of Medicine, Bronx, NY, USA.
[3] Department of Neurology, Albert Einstein College of Medicine, Bronx, NY, USA.
[4] Department of Neuroscience, Albert Einstein College of Medicine, Bronx, NY, USA.
[5] Departments of Obstetrics and Gynecology; and Pediatrics, Albert Einstein College of Medicine, Bronx, NY, USA.

* Corresponding authors

	No. of embryos	Somites	Cell viability	Number of detected cells	Mean reads/cell	Median genes/cell	Dissection
E8.5 *Wnt1-Cre/+;ROSA-EGFPf/+*	3	10-11	92%	5,987	67,537	4,088	Rostral half and heart
E9.5 *Wnt1-Cre/+;ROSA-EGFPf/+*	3	23-24	79%	9,173	42,087	4,255	Pharyngeal region (PA1-3), heart and NT
E10.5 *Wnt1-Cre/+;ROSA-EGFPf/+ #1*	3	34-35	86%	9,514	47,333	3,977	Pharyngeal region (PA2-6), heart and NT
E10.5 *Wnt1-Cre/+;ROSA-EGFPf/+ #2*	4	34-36	91%	12,047	37,306	3,723	Pharyngeal region (PA2-6), heart and NT
E9.5 *Wnt1-Cre/+;Tbx1-/-;ROSA-EGFPf/+*	3	23-25	96%	11,301	37,253	4,089	Pharyngeal region (with PA1), heart and NT
E10.5 *Wnt1-Cre/+;Tbx1-/-;ROSA-EGFPf/+ #1*	2	32-33	84%	9,918	43,353	4,512	Pharyngeal region (excluding PA1), heart and NT
E10.5 *Wnt1-Cre/+;Tbx1-/-;ROSA-EGFPf/+ #2*	2	35	80%	7,922	53,526	4,319	Pharyngeal region (excluding PA1), heart and NT

NT is neural tube, PA is pharyngeal arch

Biological replicates for E10.5 *Wnt1-Cre/+;ROSA-EGFPf/+* (#1 and #2) and E10.5 *Wnt1-Cre/+;Tbx1-/-;ROSA-EGFPf/+* (#1 and #2) were performed.

Supplementary Table 1: Summary of scRNA-seq experiments. This is summary of the scRNA-seq experiments shown in Figures 1, 2, 3, 4, 6 and 9.

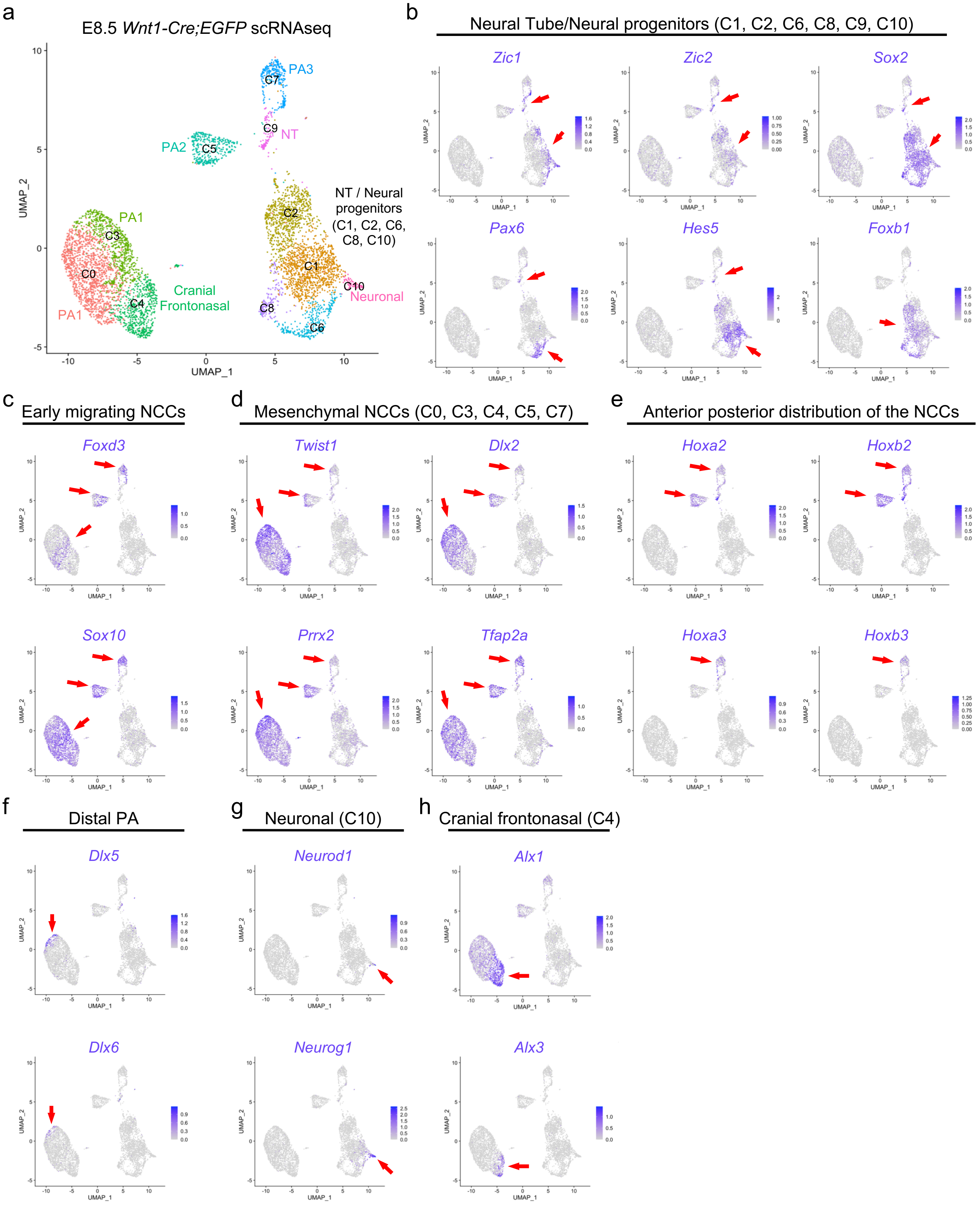

Supplementary Figure 1: Cluster identification of single cell RNA-seq data of NCCs at E8.5.
a) Seurat UMAP plot with cluster annotation of scRNA-seq data at E8.5. **b-h**) UMAP plots showing expression of specific genes, with highest expression in blue and lowest in gray, in clusters corresponding to neural tube/neural progenitors (**b**) early migrating NCCs (**c**) mesenchymal NCCs (**d**) distal part of the pharyngeal arches (**f**) neuronal cells (**g**) and cranial NCCs in the frontonasal region of embryos (**h**). Anterior posterior distribution of the NCCs in embryos were determined based on specific *Hox* genes expression (**e**). PA, pharyngeal arch; NT, neural tube.

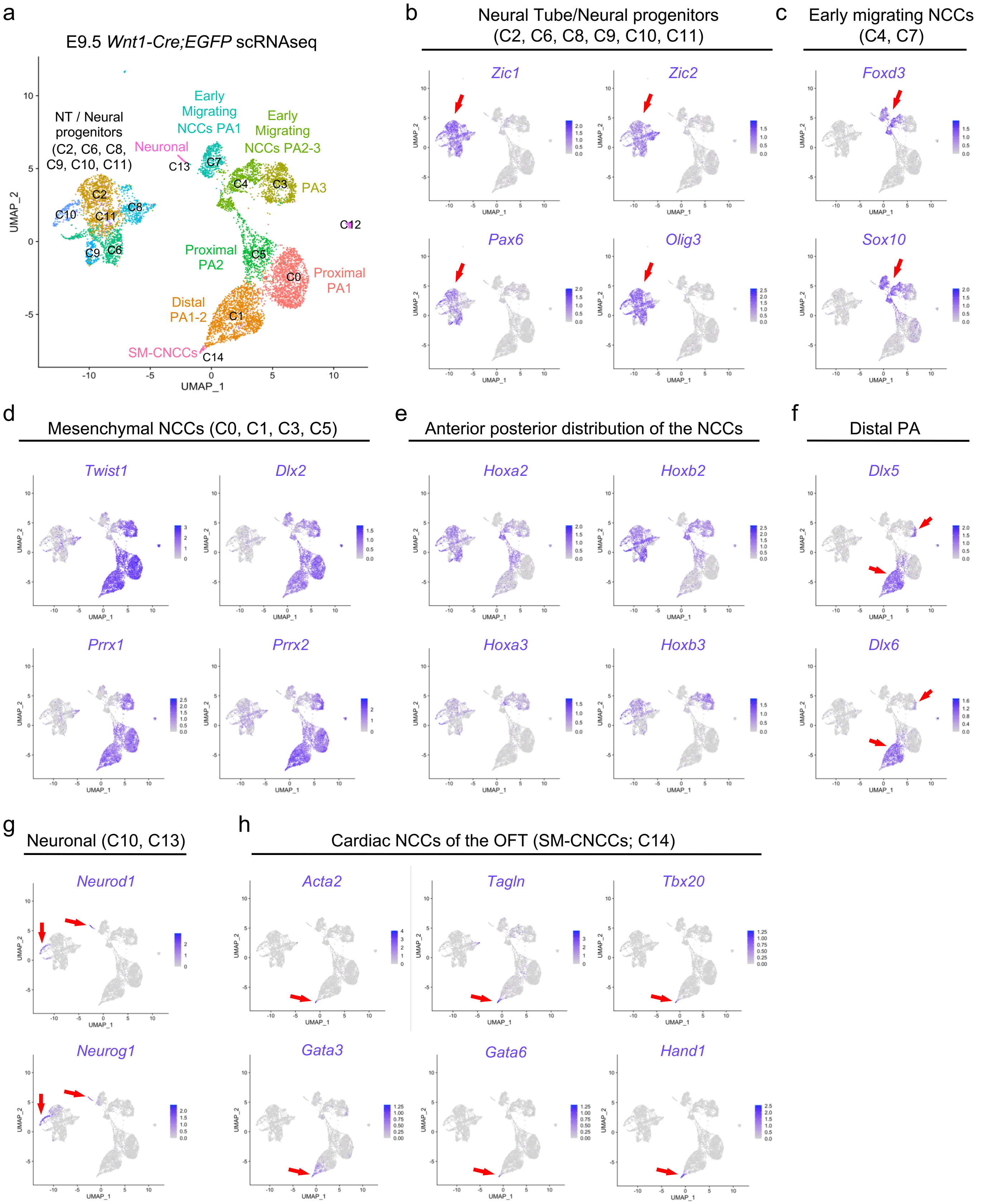

Supplementary Figure 2: Cluster identification of single cell RNA-seq data of NCCs at E9.5.
a) Seurat UMAP plot with cluster annotation of scRNA-seq data at E9.5. **b-h**) UMAP plots showing expression of specific genes, with highest expression in blue and lowest in gray, in clusters corresponding to neural tube/neural progenitors (**b**) early migrating NCCs (**c**) mesenchymal NCCs (**d**) distal part of the pharyngeal arches (**f**) neuronal cells (**g**) and cardiac NCCs in the OFT of embryos (**h**). Anterior posterior distribution of the NCCs in embryos are determined based on specific *Hox* genes expression (**e**). PA, pharyngeal arch; NT, neural tube; OFT, outflow tract; SM-CNCCs, smooth muscle cardiac neural crest cells.

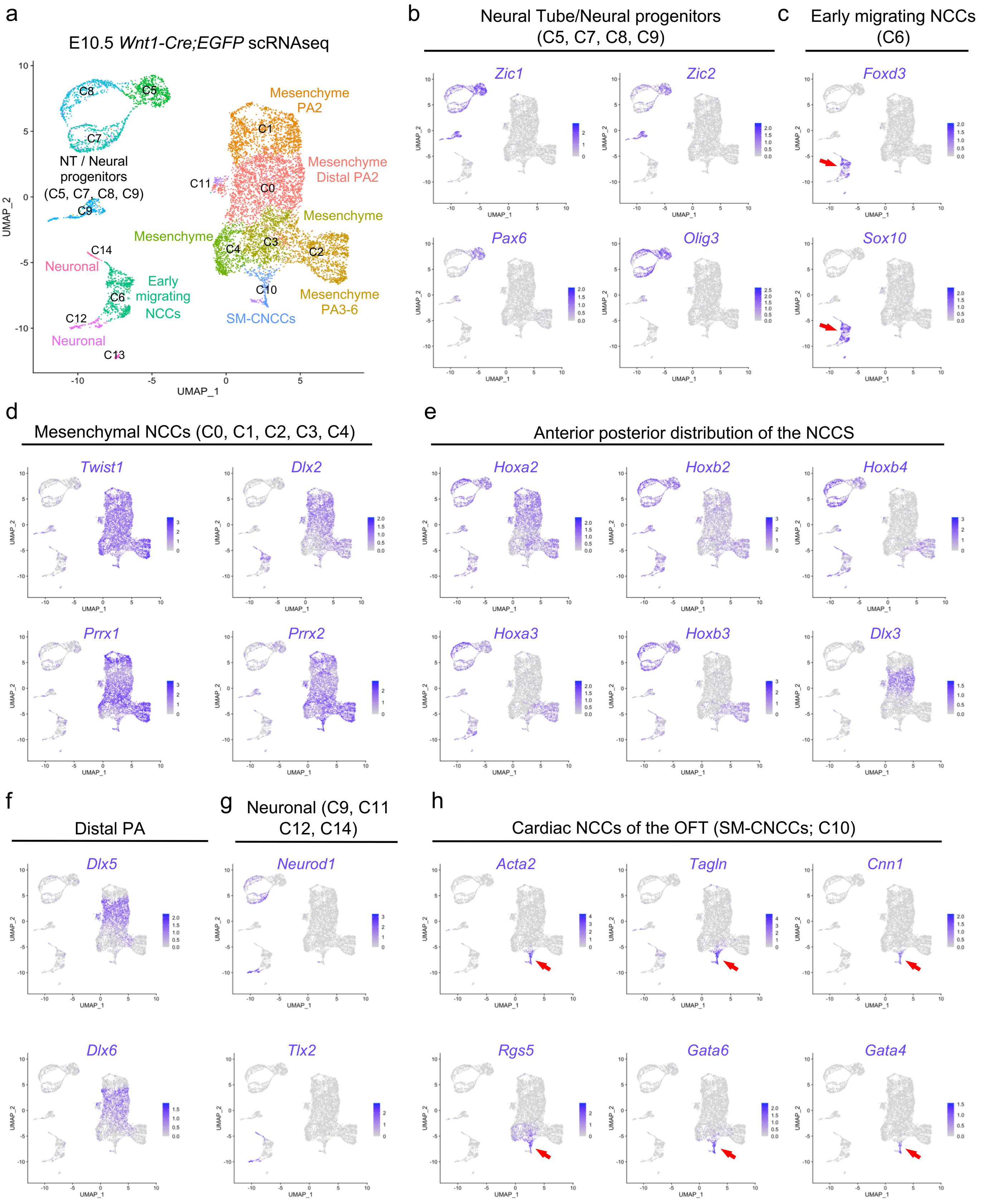

Supplementary Figure 3: Cluster identification of single cell RNA-seq data of NCCs at E10.5. **a**) Seurat UMAP plot with cluster annotation of scRNA-seq data at E10.5. B-H) UMAP plots showing expression of specific genes, with highest expression in blue and lowest in gray, in clusters corresponding to neural tube/neural progenitors (**b**) early migrating NCCs (**c**) mesenchymal NCCs (**d**) distal part of the pharyngeal arches (**f**) neuronal cells (**g**) and cardiac NCCs in the OFT of the embryo (**h**). Anterior posterior distribution of the NCCs in embryos are determined based on specific *Hox* genes expression (**e**). PA, pharyngeal arch; NT, neural tube; OFT, outflow tract; SM-CNCCs, smooth muscle cardiac neural crest cells.

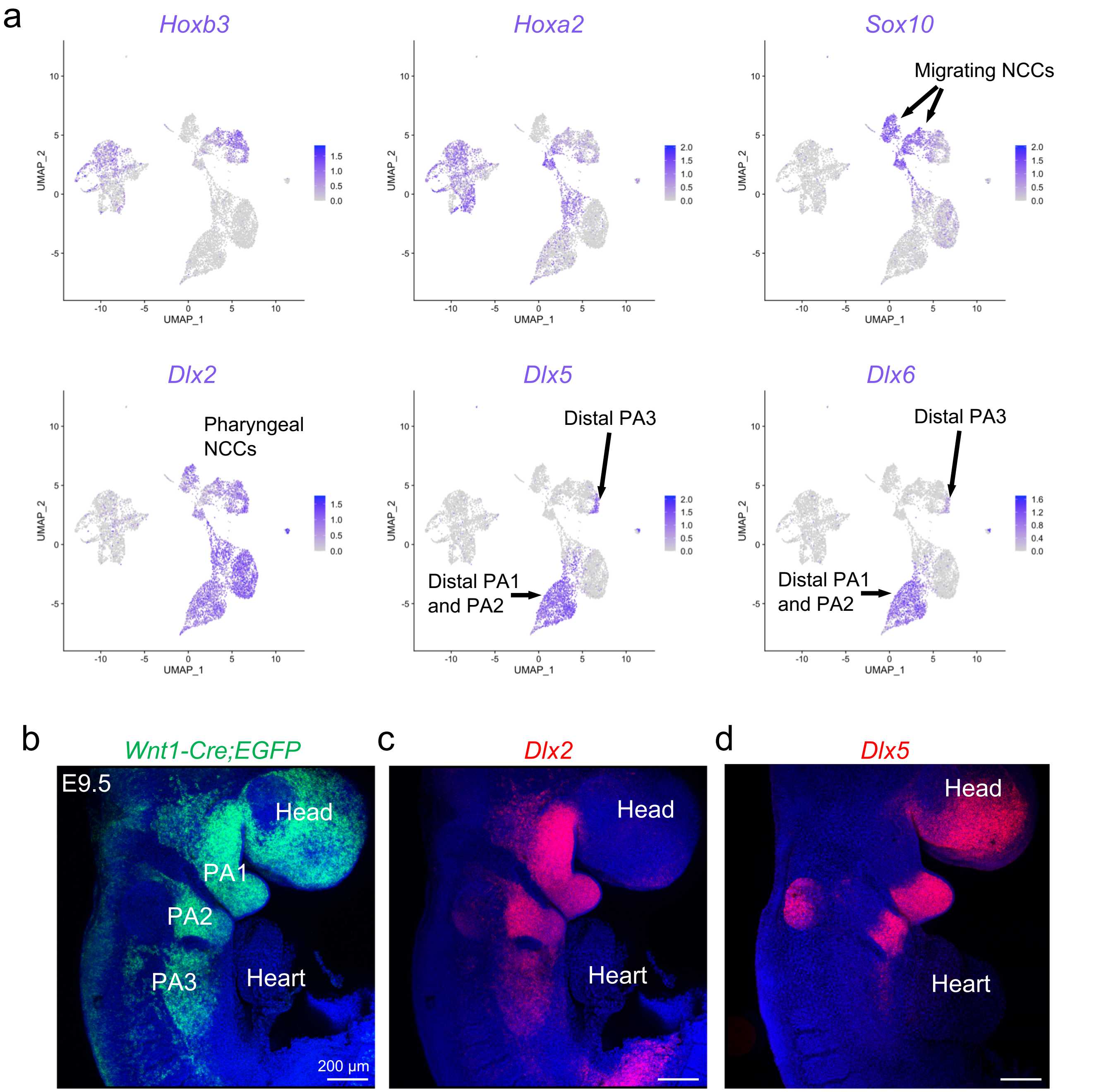

Supplementary Figure 4: *Dlx* and *Hox* genes provide proximal-distal and anterior-posterior identities of NCCs in the pharyngeal arches, respectively.
a) UMAP plots showing expression levels of *Hoxb3*, *Hoxa2*, *Sox10*, *Dlx2*, *Dlx5* and *Dlx6* genes in cell specific clusters in scRNA-seq data of NCCs at E9.5. **b-c**) Wholemount RNAscope *in situ* hybridization of *Wnt1-Cre;ROSA-EGFP* embryos at E9.5 with probes for *Egfp* and *Dlx2* (n=3). **d**) Wholemount RNAscope *in situ* hybridization of embryos at E9.5 for *Dlx5* expression (n=4). PA, pharyngeal arch. Scale bar: 200 µm. This figure is related to Figure 1.

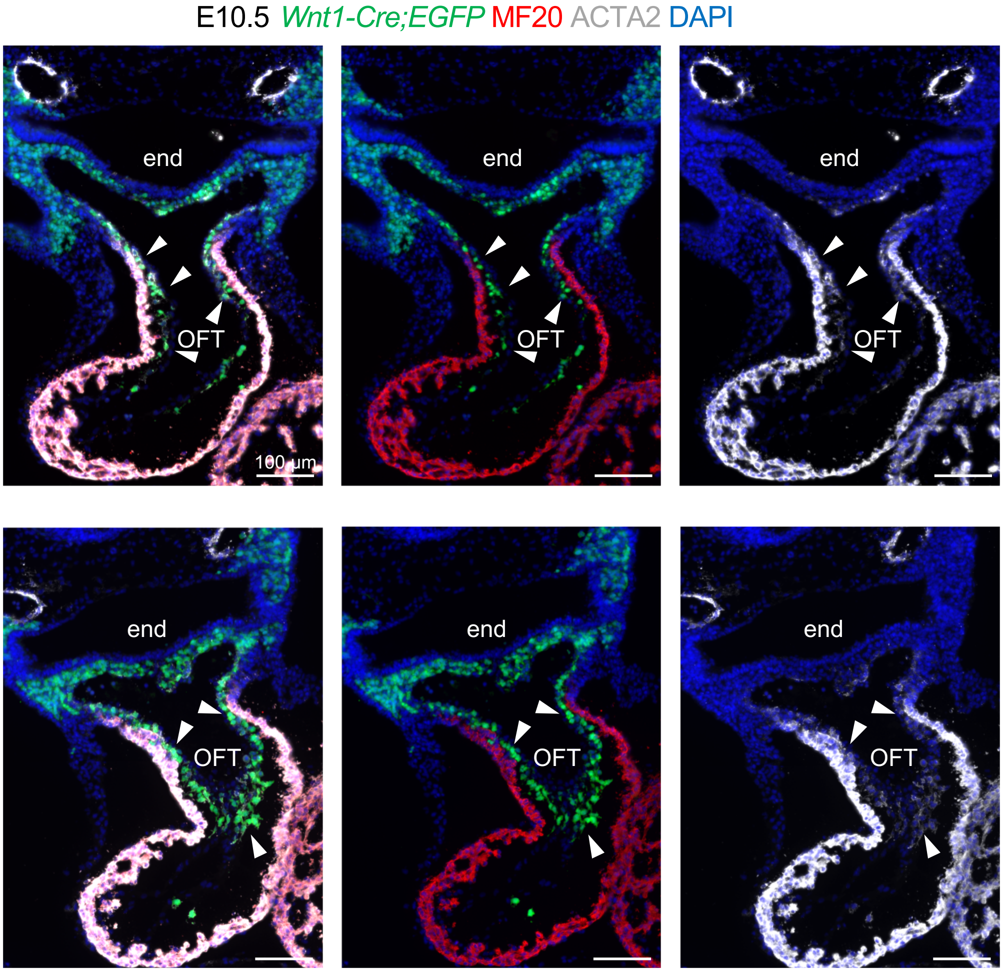

Supplementary Figure 5: Distinction between cardiac skeletal muscle progenitor cells and CNCCs in the OFT expressing ACTA2.
Immunostaining on transverse sections through *Wnt1-Cre;ROSA-EGFP* embryos at E10.5, at different anterior-posterior levels of the OFT, showing *Wnt1-Cre* genetic lineage (green), ACTA2 protein expression (gray) and cardiac skeletal muscle cells (red, MF20) (n=3). Nuclei (blue) are labelled with DAPI. Note that MF20 was not detected in CNCCs expressing ACTA2 in the OFT (arrowheads). end, endoderm; OFT, outflow tract. Scale bar: 100 µm.

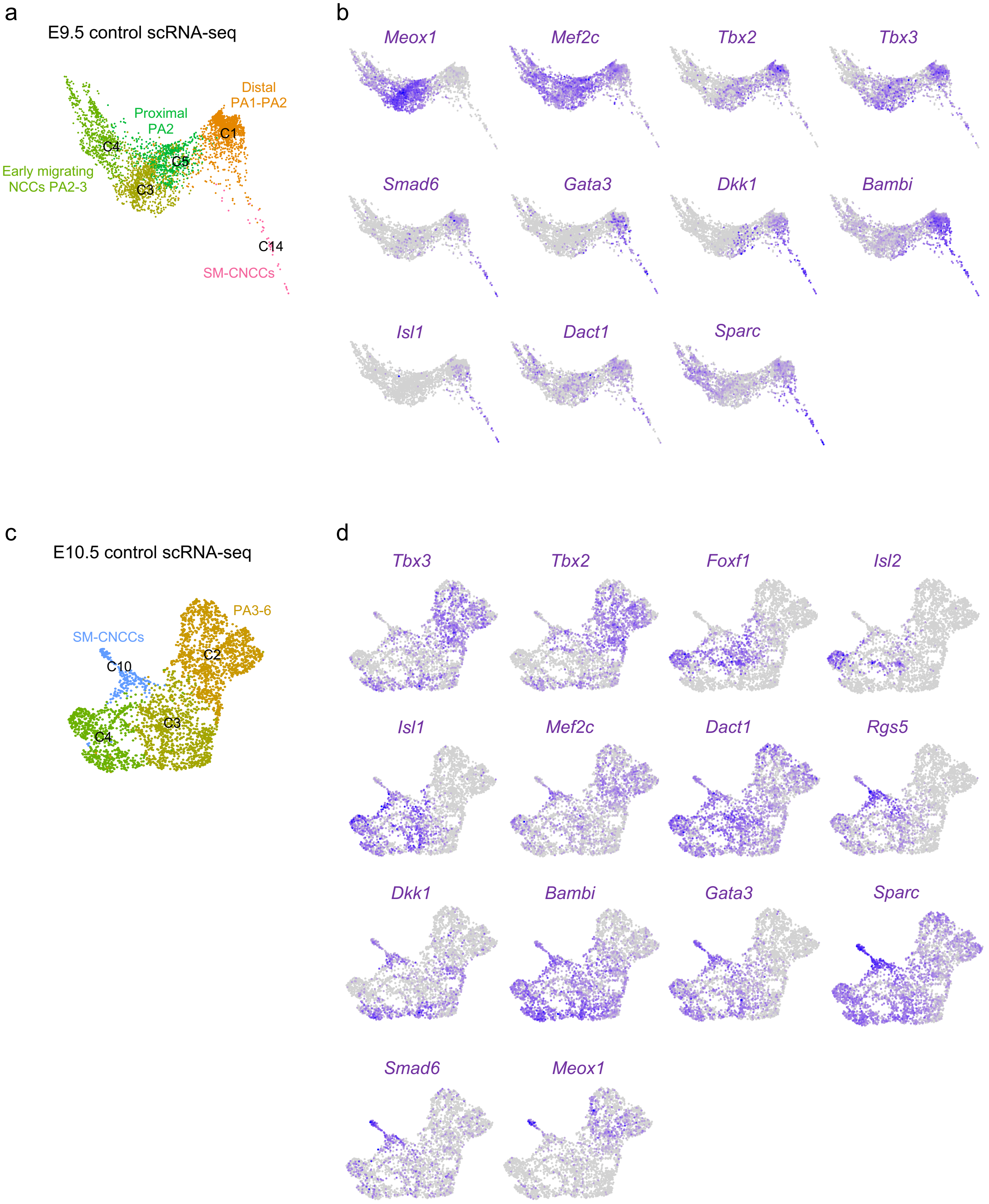

Supplementary Figure 6: Additional feature plots for genes identified in analysis of transcriptional dynamics of cardiac NCCs at E9.5 and E10.5.
a) PHATE map of NCCs in clusters C1, C3, C4, C5 and C14 of control scRNA-seq data at E9.5 using Louvain clustering with cluster annotations. **b**) PHATE maps with expression of genes at E9.5. **a** and **b** are related to Figure 2. **c**) UMAP plots of NCCs in clusters C2, C3, C4 and C10 of control scRNA-seq data at E10.5. **d**) UMAP plots with expression of marker genes at E10.5. **c** and **d** are related to Figure 3.

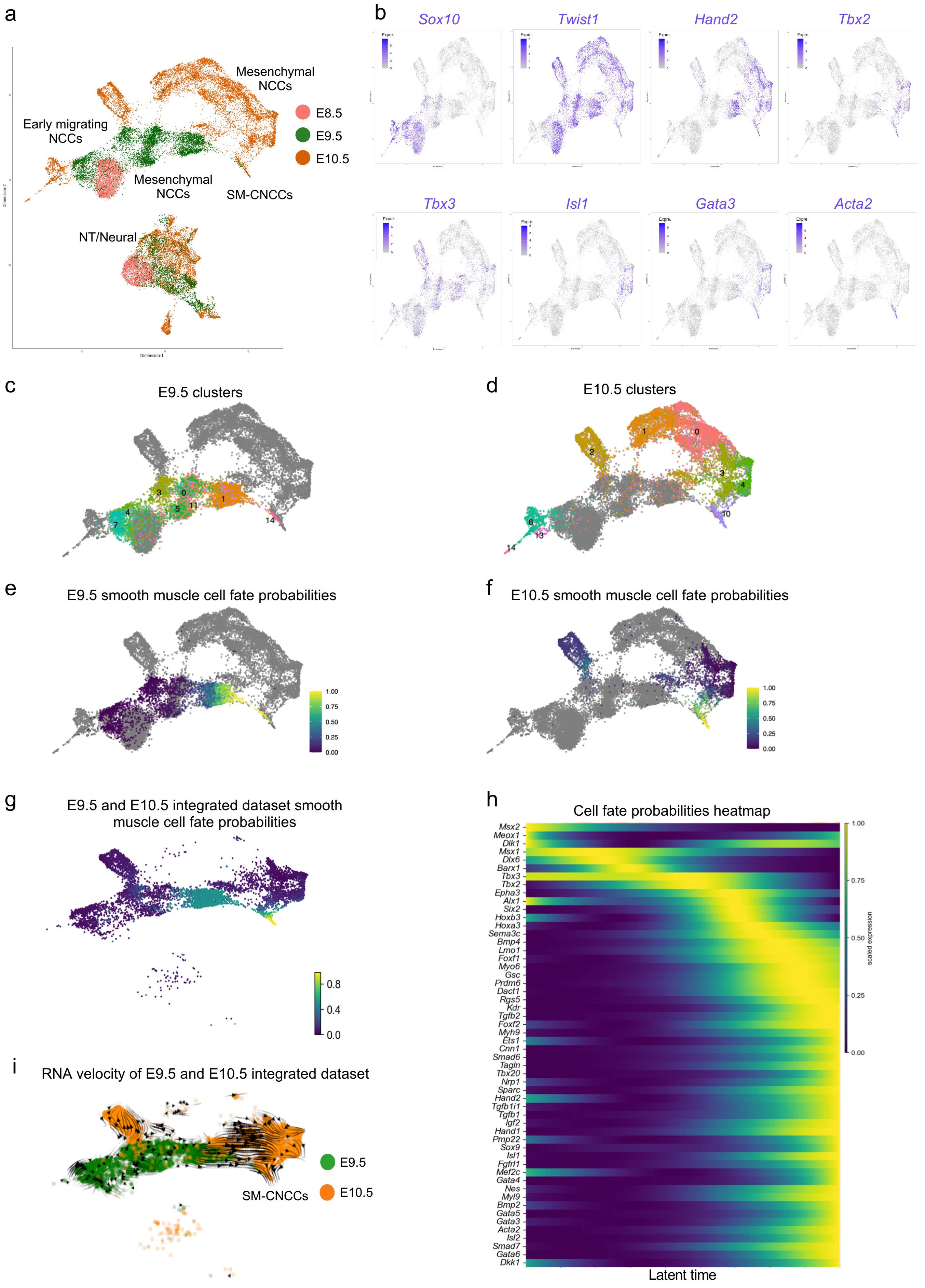
a
Mesenchymal NCCs
Early migrating NCCs
E8.5
E9.5
E10.5
Mesenchymal NCCs
SM-CNCCs
NT/Neural
Dimension-1
Dimension-2
b
Sox10
Twist1
Hand2
Tbx2
Tbx3
Isl1
Gata3
Acta2
c
E9.5 clusters
d
E10.5 clusters
e
E9.5 smooth muscle cell fate probabilities
1.00
0.75
0.50
0.25
0.00
f
E10.5 smooth muscle cell fate probabilities
1.00
0.75
0.50
0.25
0.00
g
E9.5 and E10.5 integrated dataset smooth muscle cell fate probabilities
0.8
0.4
0.0
h
Cell fate probabilities heatmap
Msx2
Meox1
Dlk1
Msx1
Dlx6
Barx1
Tbx3
Tbx2
Epha3
Alx1
Six2
Hoxb3
Hoxa3
Sema3c
Bmp4
Lmo1
Foxf1
Myo6
Gsc
Prdm6
Dact1
Rgs5
Kdr
Tgfb2
Foxf2
Myh9
Ets1
Cnn1
Smad6
Tagln
Tbx20
Nrp1
Sparc
Hand2
Tgfb1i1
Tgfb1
Igf2
Hand1
Pmp22
Sox9
Isl1
Fgfrl1
Mef2c
Gata4
Nes
Myl9
Bmp2
Gata5
Gata3
Acta2
Isl2
Smad7
Gata6
Dkk1
Latent time
1.00
0.75
0.50
0.25
0.00
scaled expression
i
RNA velocity of E9.5 and E10.5 integrated dataset
E9.5
E10.5
SM-CNCCs

Supplementary Figure 7: Cardiac smooth muscle cells fate trajectory of integrated scRNA-seq datasets of NCCs from control embryos at E8.5, E9.5 and E10.5.

a) UMAP plot of the RISC integrated scRNA-seq datasets of NCCs from *Wnt1-Cre;ROSA-EGFP* embryos at E8.5, E9.5 and E10.5 colored by developmental stages. **b**) RISC UMAP plots of the integrated scRNA-seq datasets showing expression of key genes, with highest expression in blue and lowest expression in gray. **c**, **d**) UMAP plots of the integrated scRNA-seq datasets (excluding neural cells) colored by cell clusters in pre-integrated E9.5 (**c**) or E10.5 (**d**). **e**,**f**) UMAP plots of the integrated scRNA-seq datasets (excluding neural cells) colored by smooth muscle cell fate probabilities inferred from pre-integrated E9.5 (**e**) or E10.5 (**f**) scRNA-seq data by CellRank (yellow represents high cell fate probabilities). **g**) UMAP plot of smooth muscle cell fate probabilities inferred from integrated E9.5 and E10.5 scRNA-seq datasets as define by CellRank (yellow represents high cell fate probabilities). Only clusters containing smooth muscle progenitor cells and pharyngeal NCCs with fate probabilities toward smooth muscle cells at E9.5 and E10.5 are shown. **h**) Heatmap from CellRank showing the expression of selected genes in integrated scRNA-seq datasets at E9.5 and E10.5, whose expression correlates with transitions of CNCCs fate probabilities, with cells ordered by smooth muscle fate probabilities as latent times. **i**) UMAP plot of the integrated scRNA-seq datasets at E9.5 and E10.5 with projection of RNA velocity. The arrows represent the transition probabilities of NCCs from one state to another cell state. Only clusters containing smooth muscle progenitor cells and pharyngeal NCCs with fate probabilities toward smooth muscle cells at E9.5 and E10.5 are shown.

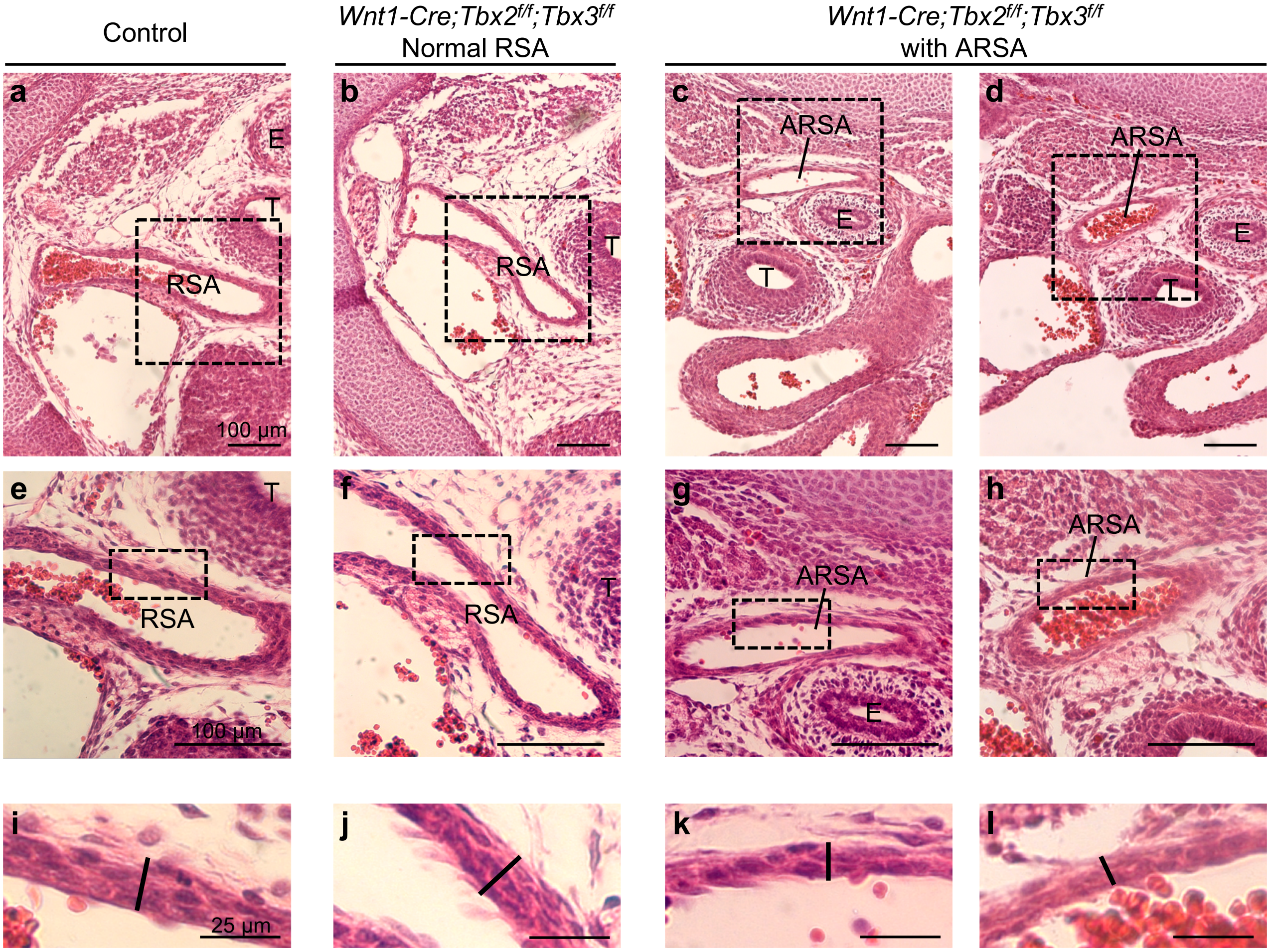

Supplementary Figure 8: Reduction of the thickness and number of smooth muscle layers in the aberrant right subclavian artery of *Wnt1-Cre;Tbx2$^{f/f}$;Tbx3$^{f/f}$* embryos at E15.5.

a-**l**) Hematotoxin and Eosin staining on transverse sections of control embryos (**a**,**e**,**i**) (n=3), *Wnt1-Cre;Tbx2$^{f/f}$;Tbx3$^{f/f}$* embryos with normal right subclavian artery (**b**,**f**,**j**) (n=3) and *Wnt1-Cre;Tbx2$^{f/f}$;Tbx3$^{f/f}$* embryos with aberrant right subclavian artery (**c**,**d**,**g**,**h**,**k**,**l**) (n=3). Panels **e**, **f**, **g** and **h** are high magnification of the dashed regions in **a**, **b**, **c** and **d**, respectively. Panels **i**, **j**, **k** and **l** are high magnification of the dashed regions in **e**, **f**, **g** and **h**, respectively. Note the qualitative reduction in the thickness of the wall of the aberrant right subclavian artery and the reduced number of smooth muscle layers in double *Tbx2/3* cKO embryos compared with control and double *Tbx2/3* cKO embryos with normal right subclavian artery (black lines in **i**, **j**, **k** and **l**). E, esophagus; T, trachea; RSA, right subclavian artery; ARSA, aberrant right subclavian artery. Scale bar: 100 µm in A-H and 25 µm in I-L.

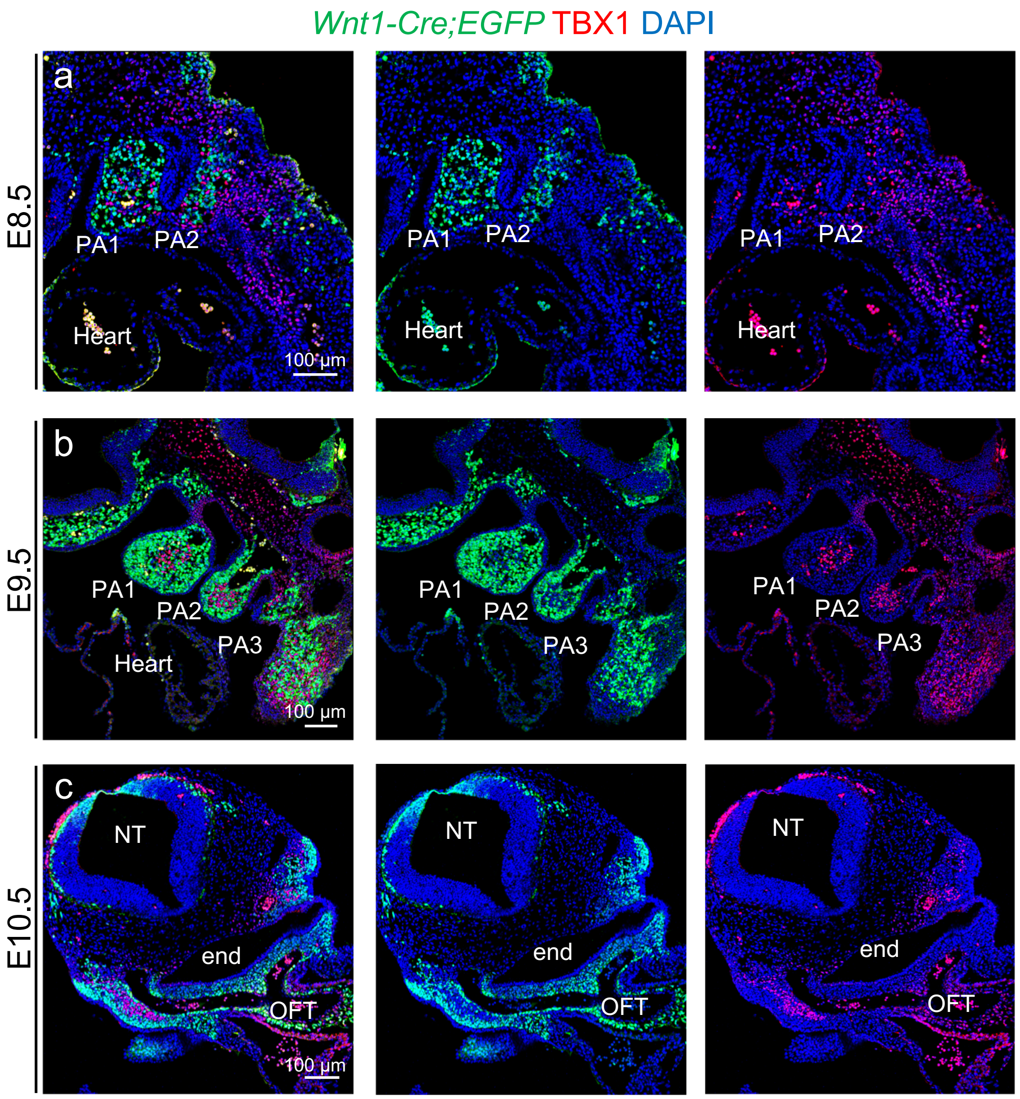

Supplementary Figure 9: TBX1 expression is not detected in NCCs at E8.5, E9.5 and E10.5. Immunostaining for EGFP (green) and TBX1 (red) on sagittal sections of *Wnt1-Cre;ROSA26-EGFP* embryos E8.5 (**a**) (n=3) and E9.5 (**b**) (n=6) and on transverse sections of *Wnt1-Cre;ROSA26-EGFP* embryos at E10.5 (**c**) (n=5). Note that TBX1 is not noticeably expressed in NCCs, but it is expressed in adjacent mesodermal cells. PA, pharyngeal arch; NT, neural tube; end, endoderm; OFT, outflow tract. Scale bars: 100 µm.

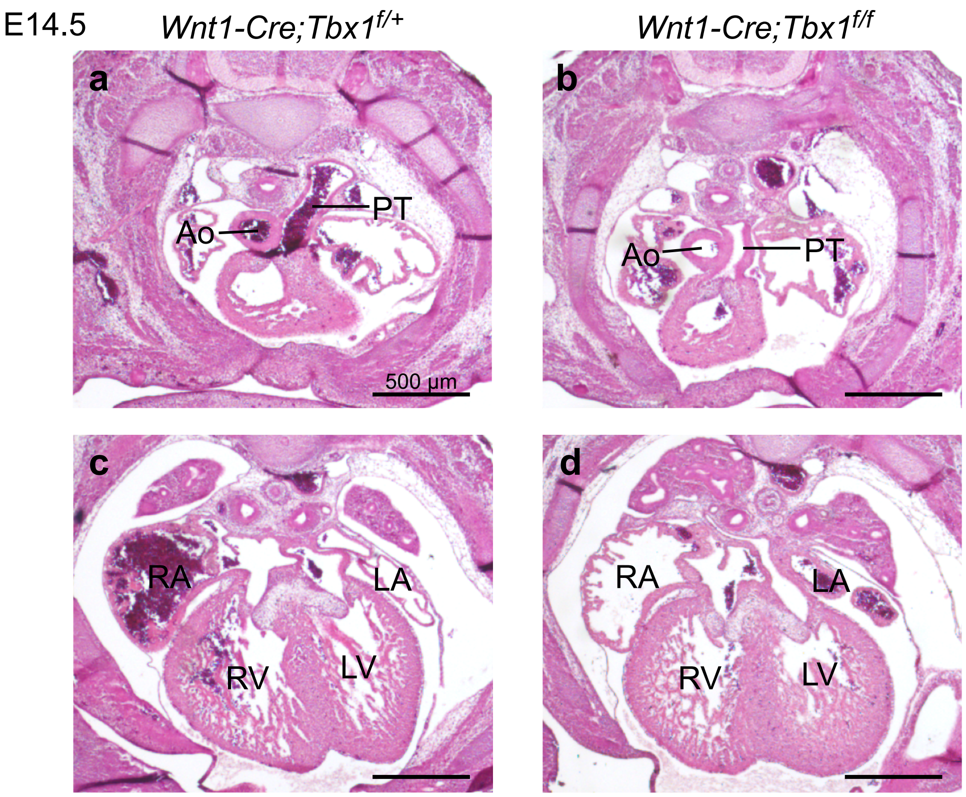

Supplementary Figure 10: Conditional deletion of *Tbx1* in NCCs does not affect heart development.

Hematoxylin and eosin staining on *Wnt1-Cre;Tbx1*$^{f/+}$ (n=3) and *Wnt1-Cre;Tbx1*$^{f/f}$ (n=3) embryos at E14.5 showing normal septation of the aorta and pulmonary trunk (**a**,**b**) and normal interventricular septation in *Wnt1-Cre;Tbx1*$^{f/f}$ embryos (**c**,**d**). Ao, aorta; PT, pulmonary trunk; RA, right atrium; LA, left atrium; RV, right ventricle; LV, left ventricle. Scale bars: 500 µm.

a

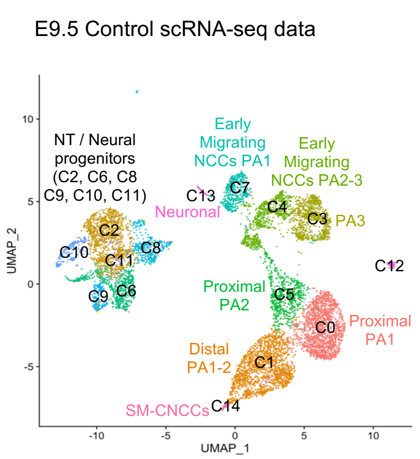

b

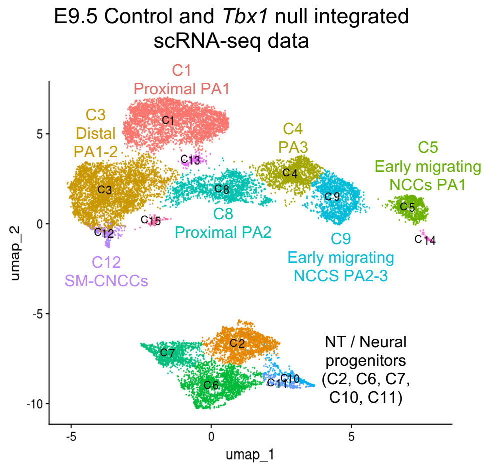

c

Control \ Integrated	C1	C2	C3	C4	C5	C6	C7	C8	C9	C10	C11	C12	C13	C14	C15
C0	**1531**	0	29	3	6	0	0	20	1	0	2	0	2	0	10
C1	44	1	**1329**	2	1	1	1	149	1	0	0	72	0	0	2
C2	0	695	0	0	0	370	30	1	0	0	2	0	0	0	1
C3	2	2	4	**699**	0	1	1	37	60	0	0	0	0	0	1
C4	0	1	0	14	25	1	1	7	**672**	0	0	0	0	0	0
C5	20	0	8	16	0	1	0	**580**	16	0	0	0	2	0	0
C6	0	8	0	0	0	466	1	0	0	0	100	0	0	1	0
C7	12	2	3	0	**434**	0	0	1	12	0	0	0	0	1	1
C8	0	15	0	0	0	19	342	0	0	0	0	0	0	0	13
C9	0	3	0	0	0	171	1	0	0	0	1	0	0	0	0
C10	0	0	0	0	0	0	0	0	0	166	0	0	0	0	0
C11	9	33	24	7	0	28	13	5	7	0	3	0	0	0	1
C12	9	0	4	2	0	0	0	4	0	0	0	0	57	0	0
C13	0	0	0	0	1	0	0	0	0	0	0	0	0	12	0
C14	0	0	0	0	0	0	0	0	0	0	0	**39**	0	0	0

d

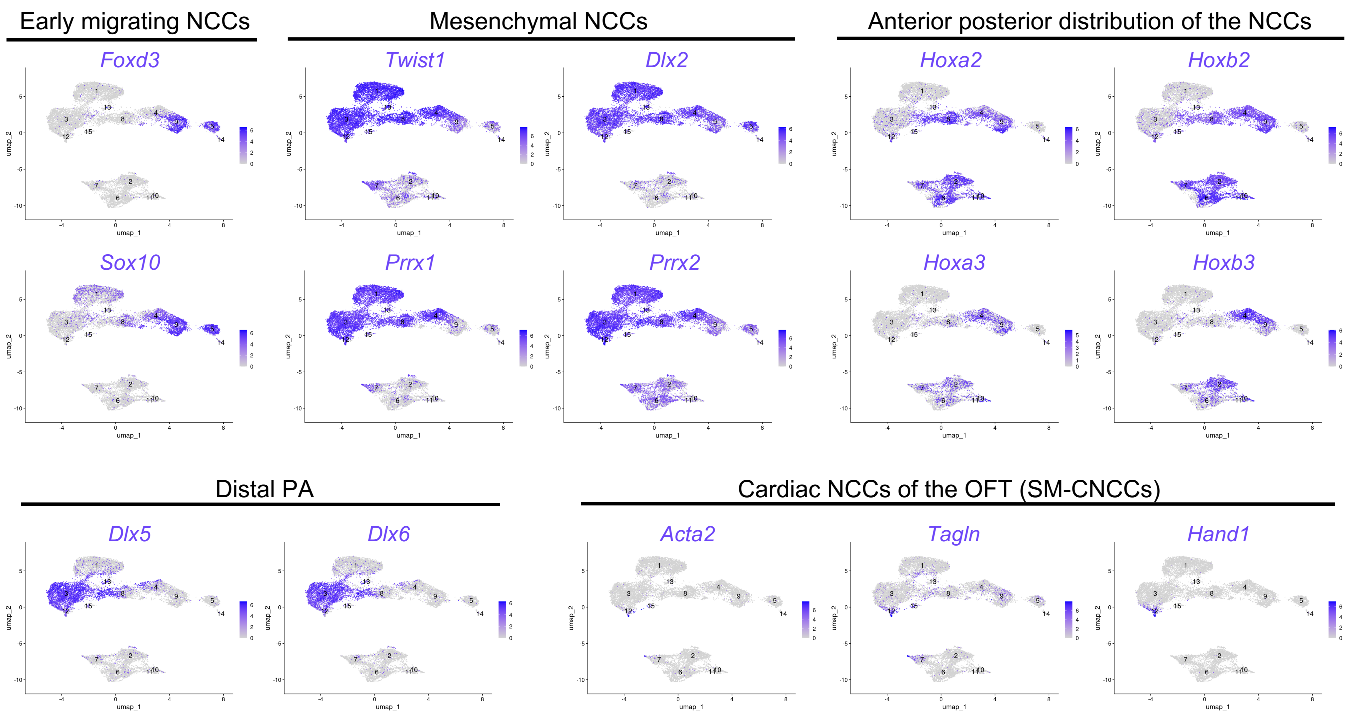

Supplementary Figure 11: Clustered cells in scRNA-seq data from control embryos are also clustered together in integrated control and *Tbx1* null scRNA-seq data analysis at E9.5.

a) Seurat UMAP plot with cluster annotation of scRNA-seq data of NCCs from control embryos at E9.5. This is related to Figure 1e. **b**) RISC UMAP plot with cluster annotation of integrated scRNA-seq data of NCCs from control and *Tbx1* null embryos at E9.5. This is related to Figure 6b. **c**) Table showing the absolute number of cells from control embryos only (based on actual barcodes) in each cluster in **a** and **b**. Rows correspond to a cluster from scRNA-seq data from controls and columns correspond to clusters from RISC integrated scRNA-seq data. Note that most of the cells in clusters identified in scRNA-seq data analysis of controls (**a**) are still clustered together in the integrated data of controls and *Tbx1* null embryos (**b**). Numbers in red color correspond to early migrating NCCs and NCCs in the pharyngeal arches and cardiac OFT. **d**) UMAP plots showing expression of genes, with highest expression in blue and lowest in gray, in clusters corresponding to early migrating NCCs, mesenchymal NCCs, distal part the pharyngeal arches and NCCs in the cardiac OFT in scRNA-seq data from control and *Tbx1* null integrated datasets. PA, pharyngeal arch; NT, neural tube; SM-CNCCs, smooth muscle NCCs.

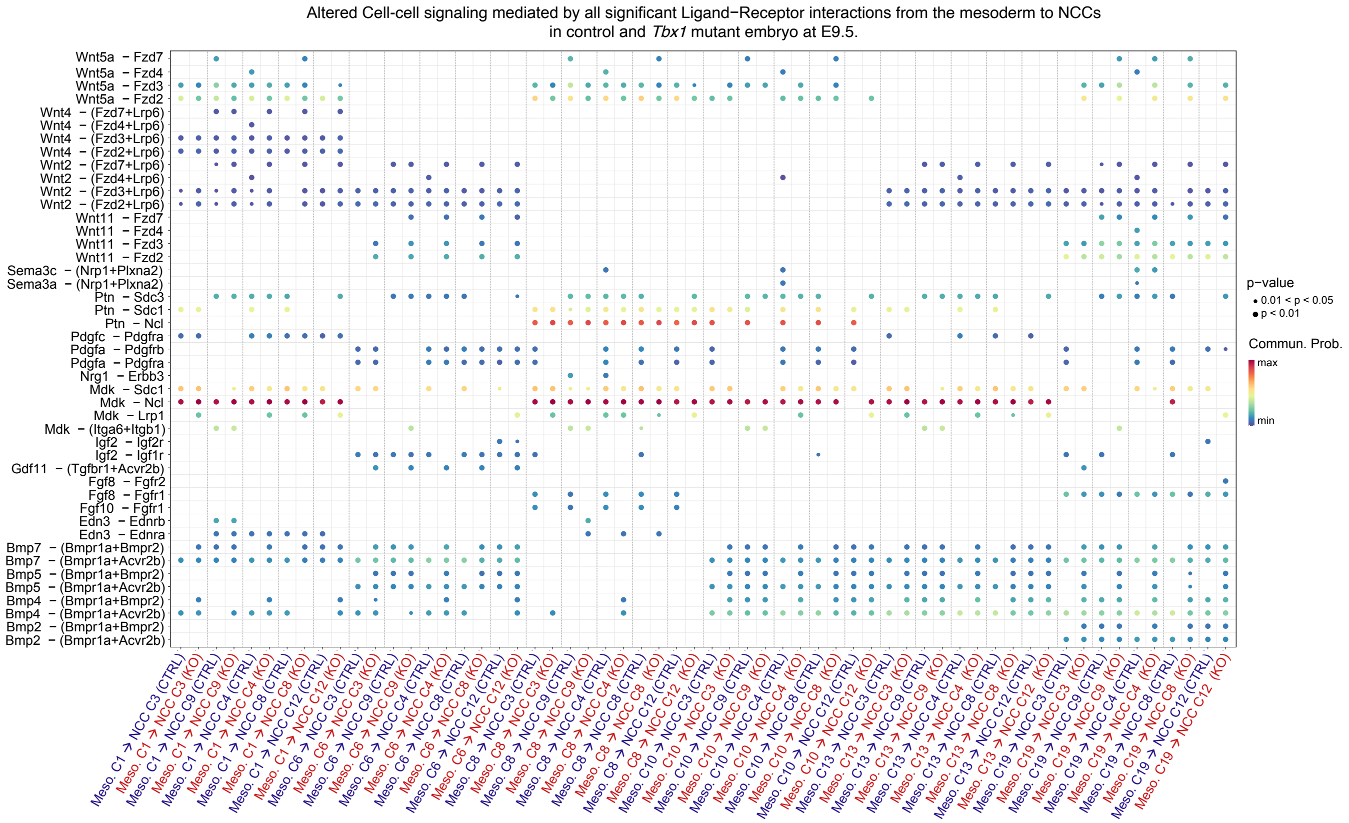

Supplementary Figure 12: Bubble plots for all ligand-receptor pairs showing significant cell-cell signaling changes from mesodermal cells to NCCs in control and *Tbx1* mutant embryos at E9.5. This version is the complete version compared to the image shown in Fig. 8b. Wilcox rank two-side test without multiple test correction was used, p-value is indicated by size of dot and color.

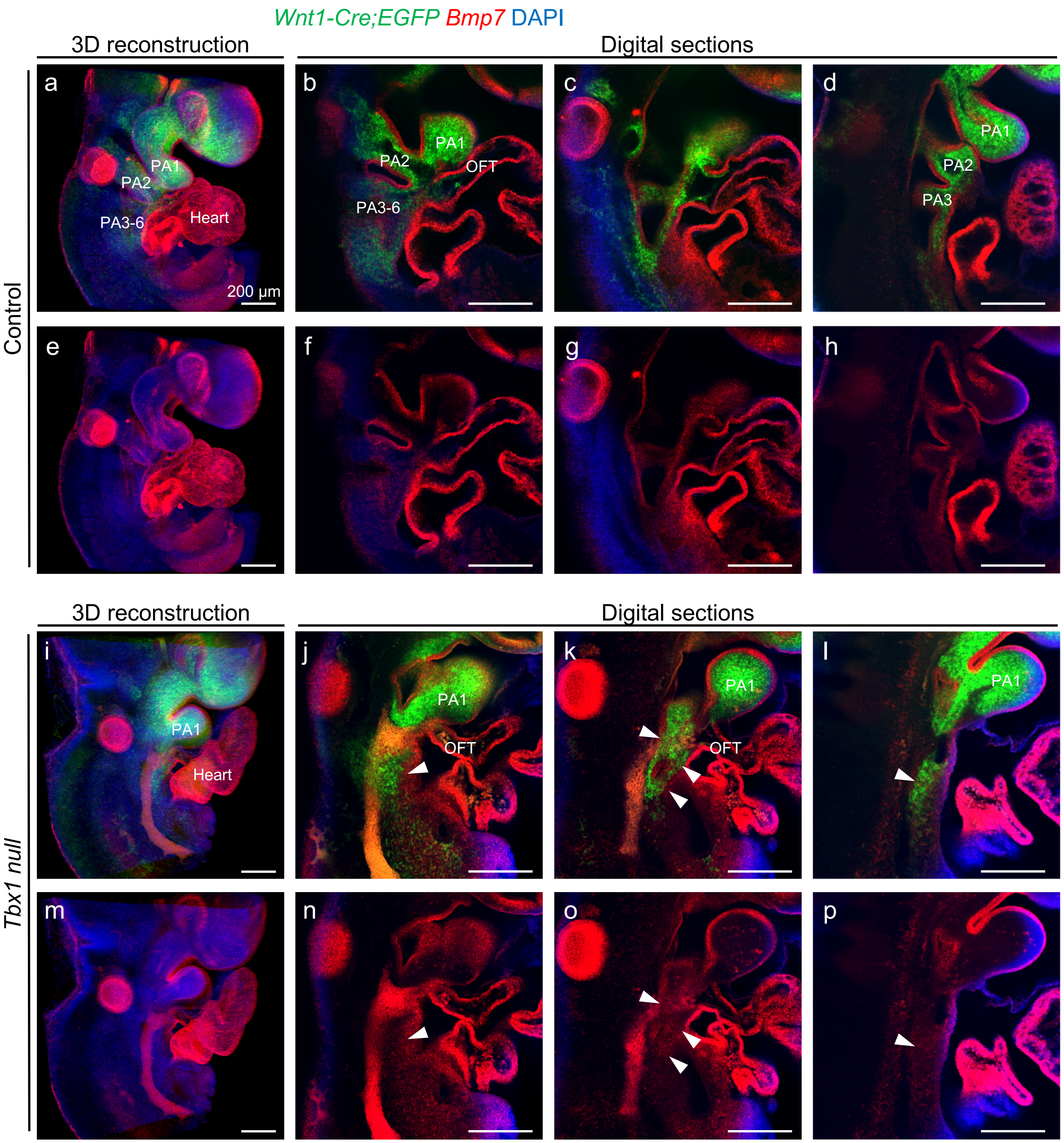

Supplementary Figure 13: *Bmp7* expression is strongly maintained in the pharyngeal apparatus of *Tbx1* null embryos at E9.5.

a-p) Wholemount RNAscope *in situ* hybridization of *Wnt1-Cre;ROSA-EGFP* control (**a-h**) and *Tbx1* null (**i-p**) embryos at E9.5 with probes for *Egfp*, and *Bmp7*. Nuclei, in blue, are stained with DAPI. 3D reconstruction of wholemount control (**a**,**e**) and *Tbx1* null (**i**,**m**) images and digital sections at different location through the left right axis of control (**b**, **c**, **d**, **f**, **g**, **h**) and *Tbx1* null (**j**, **k**, **l**, **m** ,**n**, **o**, **p**) embryos are shown. Arrowheads show the expression of *Bmp7* in cells directly adjacent to NCCs in the pharyngeal region of *Tbx1* null mutant embryos (**j**, **k**, **l**, **m**, **n**, **o**, **p**).

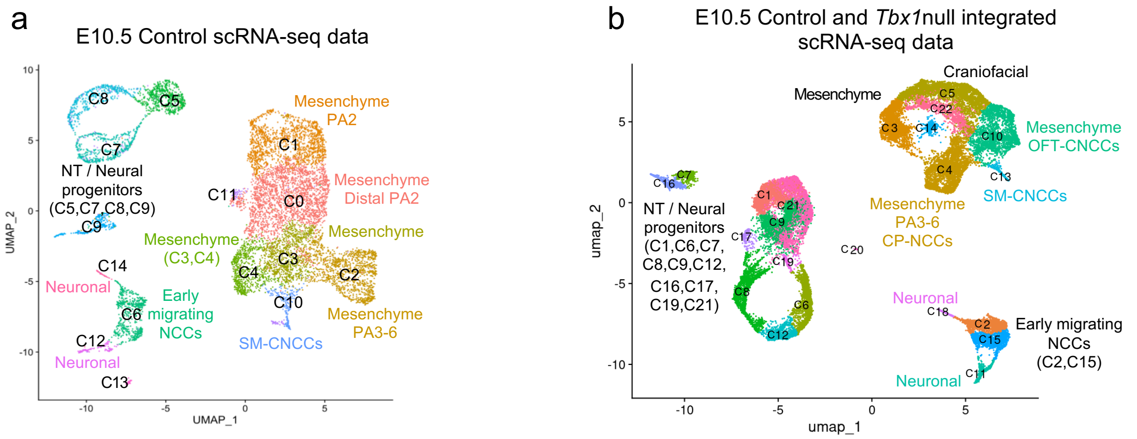

c

Control \ Integrated	C1	C2	C3	C4	C5	C6	C7	C8	C9	C10	C11	C12	C13	C14	C15	C16	C17	C18	C19	C20	C21	C22
C0	1	0	21	27	1709	0	0	0	0	36	0	0	1	77	0	0	0	0	0	0	0	455
C1	0	1	1139	23	320	1	0	0	0	2	0	0	0	7	0	0	0	0	0	0	0	21
C2	0	1	16	**1208**	27	0	0	0	0	16	0	0	0	5	0	0	0	0	0	0	0	72
C3	3	0	3	29	283	0	0	0	0	**688**	0	0	2	2	0	0	0	0	0	0	0	15
C4	1	0	4	7	19	0	0	0	0	**714**	0	0	0	0	0	0	0	0	0	0	0	3
C5	120	0	4	5	13	2	0	5	174	2	0	0	0	1	0	0	5	0	5	0	304	2
C6	0	**336**	2	5	22	1	1	1	0	8	5	0	0	1	**255**	0	0	3	0	0	0	1
C7	0	0	0	0	1	303	0	5	3	1	0	169	0	1	0	0	0	0	17	0	2	0
C8	1	0	1	0	0	0	0	418	5	2	0	4	0	0	1	0	8	0	0	0	0	0
C9	4	0	1	0	0	2	89	0	0	0	0	0	2	3	0	181	0	0	0	0	1	0
C10	0	0	1	6	0	0	0	0	0	66	0	0	**148**	2	0	0	0	0	0	0	0	0
C11	2	0	4	11	26	14	0	13	4	6	0	8	1	123	0	0	0	0	1	1	0	3
C12	0	0	0	0	0	0	0	0	0	0	118	0	0	0	1	0	0	0	0	0	0	0
C13	0	44	0	1	2	0	0	0	0	1	0	1	0	0	7	0	1	0	0	0	0	0
C14	0	0	0	0	0	0	0	0	0	0	0	2	1	2	0	0	0	40	0	0	0	0

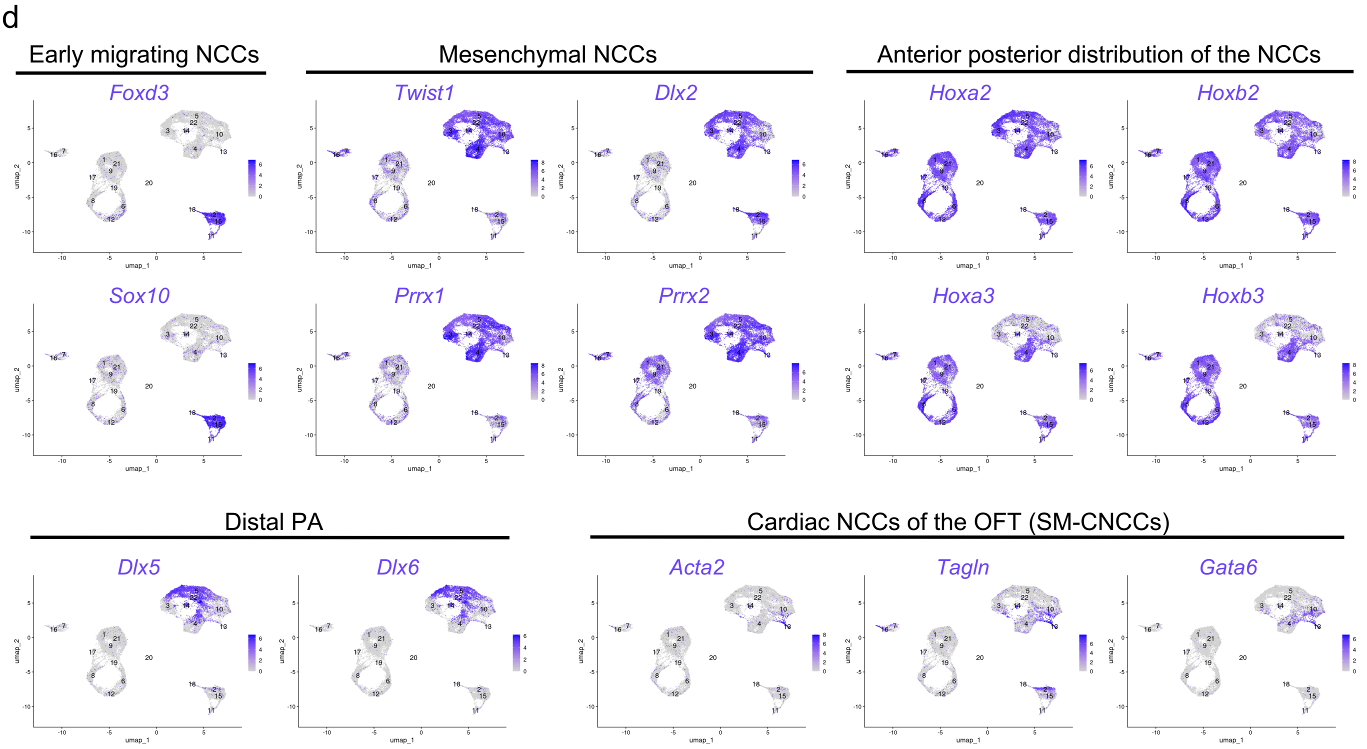

Supplementary Figure 14: Clustered cells in scRNA-seq data from control embryos are also clustered together in integrated scRNA-seq data from control and *Tbx1* null embryos at E10.5. **a**) Seurat UMAP plot with cluster annotations of scRNA-seq data of NCCs from control embryos at E10.5. This is related to Figure 1f. **b**) RISC UMAP plot with cluster annotations of integrated scRNA-seq data of NCCs from control and *Tbx1* null embryos at E10.5. This is related to Figure 9b. **c**) Table showing the absolute number of cells from control embryos only (based on actual barcodes) in each cluster in **a** and **b**. Rows correspond to clusters from scRNA-seq data in control embryos and columns correspond to clusters from RISC integrated scRNA-seq data. Note that most of the cells in clusters identified in controls (**a**) are still clustered together in the integrated control and *Tbx1* null scRNA-seq dataset (**b**). Numbers in red color correspond to early migrating NCCs, mesenchymal NCCs in PA3-6 containing the CP-NCCs and OFT-CNCCs and NCCs in the cardiac OFT. **d**) UMAP plots showing expression of genes, with highest expression in blue and lowest in gray, in clusters corresponding to early migrating NCCs, mesenchymal NCCs, distal part the pharyngeal arches and NCCs in the cardiac OFT in scRNA-seq data from controls and *Tbx1* null integrated datasets. PA, pharyngeal arch; NT, neural tube; SM-CNCCs, smooth muscle NCCs.

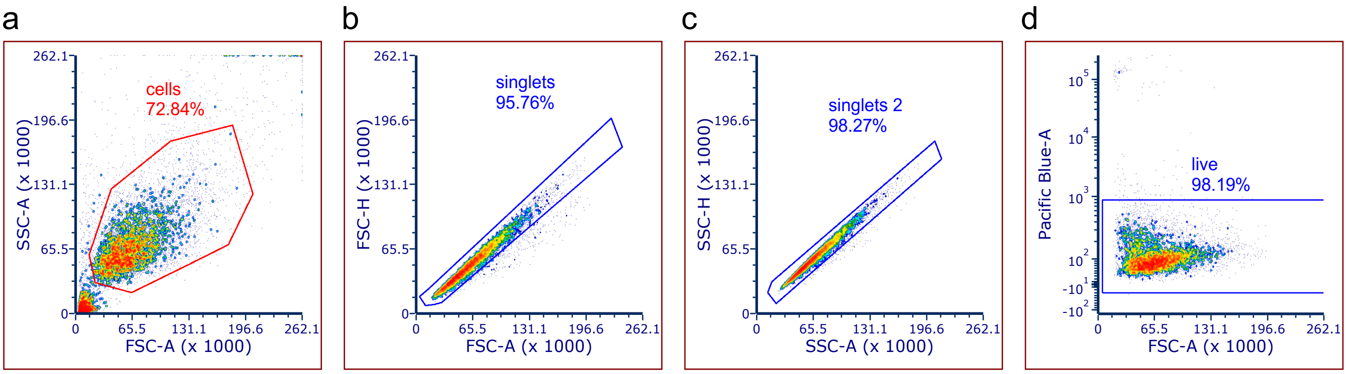

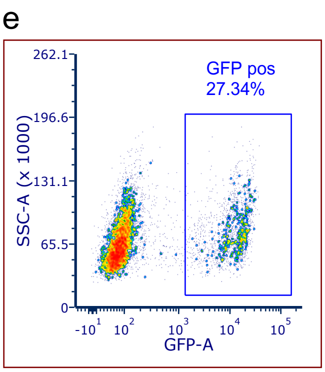

f

	Total number of FACS purified cells
E8.5 *Wnt1-Cre/+;ROSA-EGFPf/+*	23,440
E9.5 *Wnt1-Cre/+;ROSA-EGFPf/+*	52,866
E10.5 *Wnt1-Cre/+;ROSA-EGFPf/+ #1*	164,283
E10.5 *Wnt1-Cre/+;ROSA-EGFPf/+ #2*	318,578
E9.5 *Wnt1-Cre/+;Tbx1-/-;ROSA-EGFPf/+*	73,854
E10.5 *Wnt1-Cre/+;Tbx1-/-;ROSA-EGFPf/+ #1*	72,007
E10.5 *Wnt1-Cre/+;Tbx1-/-;ROSA-EGFPf/+ #2*	97,449

Supplementary Figure 15: Gating strategy for GFP positive cell sorting.
a) FSC-A/SSC-A (FSC-A, forward scatter area; SSC-A, side scatter area) plot show gated cells. **b-c**) Singlets were gated in FSC-A/FSC-H (FSC-area, FSC-height) plot (**b**) and in SSC-A/SSC-H (SSC-area; SSC-height) plot (**c**). The viable cells were gated in FSC-A/Pacific Blue-A plot (**d**). GFP positive cells were gated in GFP-A/SSC-A plot (GFP pos) (**e**). The cell population density is color coded (red, high density; blue, low density). **f**) Table summarizing the total number of GFP positive cells sorted for each experiments. This table is related to Supplementary Table 1.
