# Supplementary material for: Single-cell transcriptomics uncovers a non-autonomous Tbx1-dependent genetic program controlling cardiac neural crest cell development
Source: Nat Commun. 2023 Mar 21;14:1551. doi: 10.1038/s41467-023-37015-9 (PMC10027855; doi:10.1038/s41467-023-37015-9)
Supplement: Supplementary file 3 — Description of Additional Supplementary Files [file 41467_2023_37015_MOESM3_ESM.pdf]

Description of Additional Supplementary Files

File Name: Supplementary Data 1
Description: **Marker genes and statistics of cell clusters from scRNA-seq of NCCs at E8.5.** These data are related to Fig. 1.

File Name: Supplementary Data 2
Description: **Marker genes and statistics of cell clusters from scRNA-seq of NCCs at E9.5.** These data are related to Fig. 1 and 2.

File Name: Supplementary Data 3
Description: **Marker genes and statistics of cell clusters from scRNA-seq of NCCs at E10.5.** These data are related to Fig. 1 and 3.

File Name: Supplementary Data 4
Description: **Heatmap of gene expression that correlates with cardiac fate probabilities with cells ordered by fate probabilities at E9.5.** This heatmap is the complete version of the selected genes shown in Fig. 2.

File Name: Supplementary Data 5
Description: **Heatmap of gene expression that correlates with cardiac fate probabilities with cells ordered by fate probabilities at E10.5.** This heatmap is the complete version of the selected genes shown in Fig. 4.

File Name: Supplementary Data 6
Description: **Gene ontology biological processes and disease processes of lineage driver genes at E10.5.** These are the complete lists of gene ontology biological processes and disease processes in each group of genes after dividing the ordered gene list in Supplementary data 5 into four groups of equal number of genes from *Bmp4* to *Gata6*.

File Name: Supplementary Data 7
Description: **List of differentially expressed genes and statistics for each NCC cluster of integrated data from control and *Tbx1* null embryos at E9.5.** This list is associated with the data shown in Fig. 6b and c.

File Name: Supplementary Data 8
Description: **List of differentially expressed genes and statistics for each cell cluster of integrated data from control and *Tbx1* null embryos at E10.5.** This list is related to data shown in Fig. 9b and c.

File Name: Supplementary Data 9
Description: **Gene ontology biological processes of upregulated genes in pharyngeal NCCs (C4 in Figure 9) from control and *Tbx1* null embryos at E10.5**. This is a complete version of the data shown in Fig. 9h.

File Name: Supplementary Data 10
Description: **Gene ontology biological processes of downregulated genes in pharyngeal NCCs (C4 in Figure 9) from control and *Tbx1* null embryos at E10.5**. This is a complete version of the data shown in Fig. 9i.

File Name: Supplementary Data 11
Description: **Gene ontology biological processes of upregulated genes in OFT-CNCCs (C10 in Figure 9) from control and *Tbx1* null embryos at E10.5.** This is a complete version of the data shown in Fig. 9j.
