# Supplementary figures and images for: Single-cell transcriptomics uncovers a non-autonomous Tbx1-dependent genetic program controlling cardiac neural crest cell development
Source: Nat Commun. 2023 Mar 21;14:1551. doi: 10.1038/s41467-023-37015-9 (PMC10027855; doi:10.1038/s41467-023-37015-9)

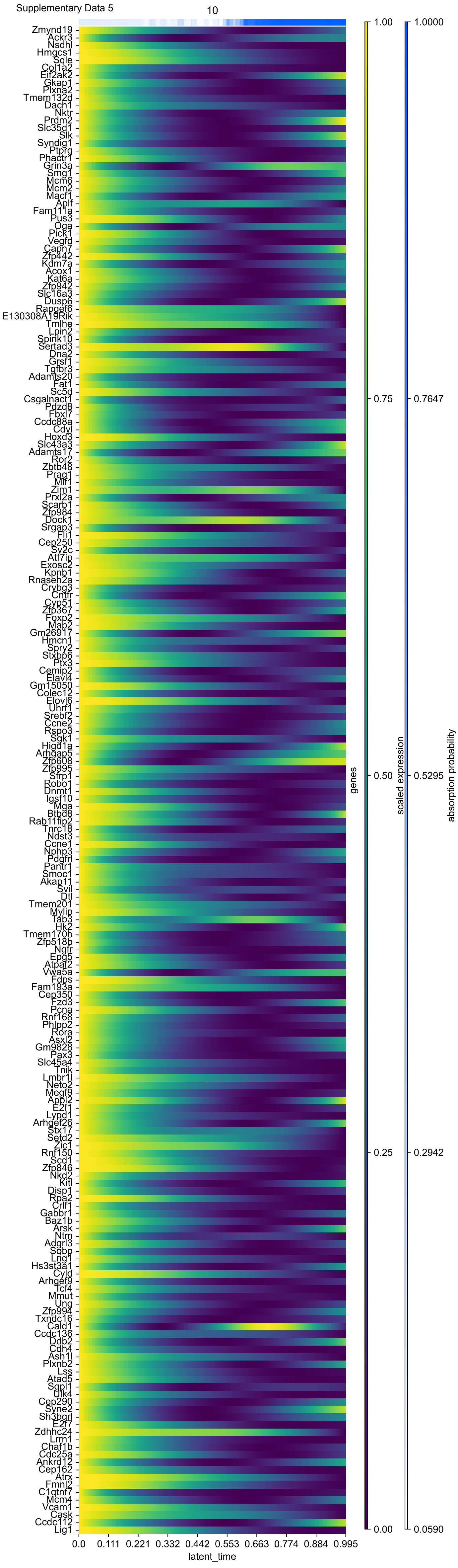

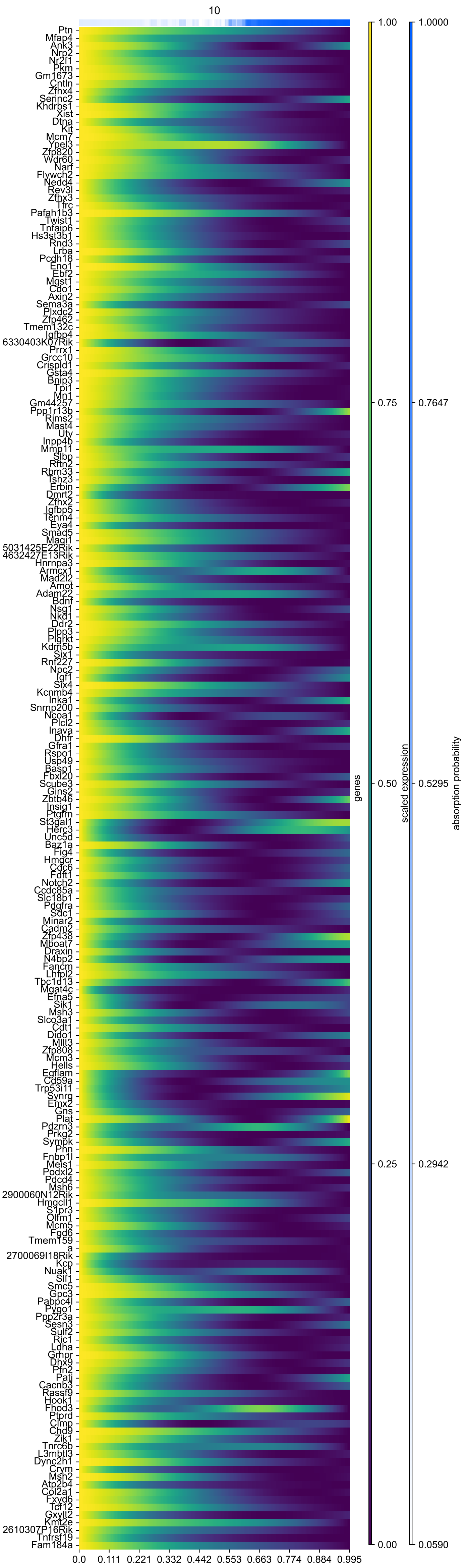

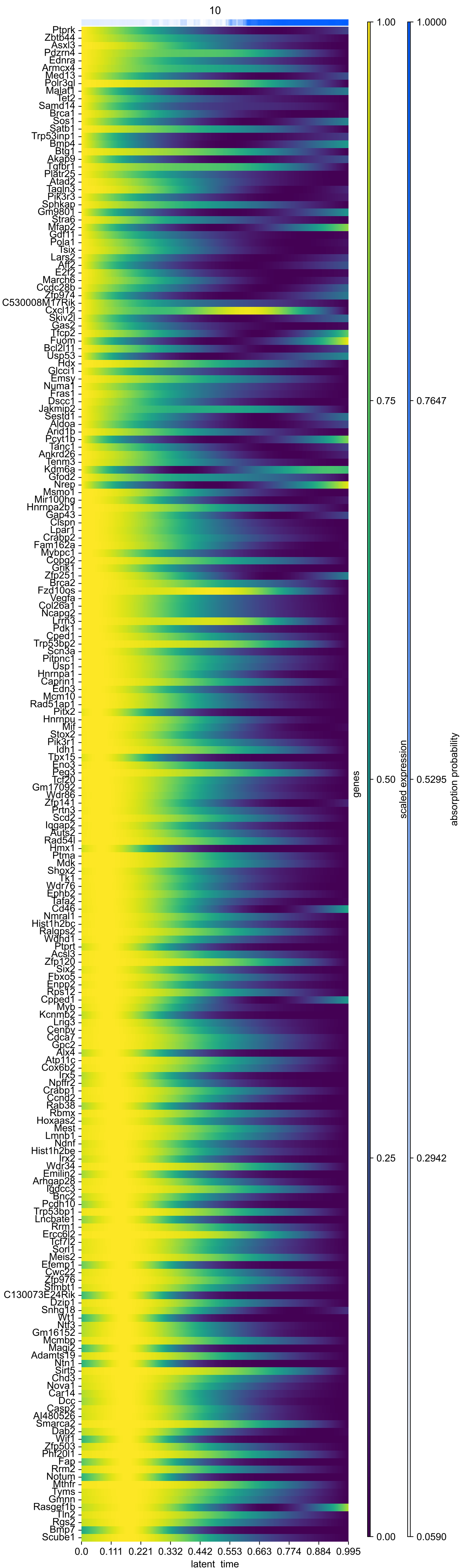

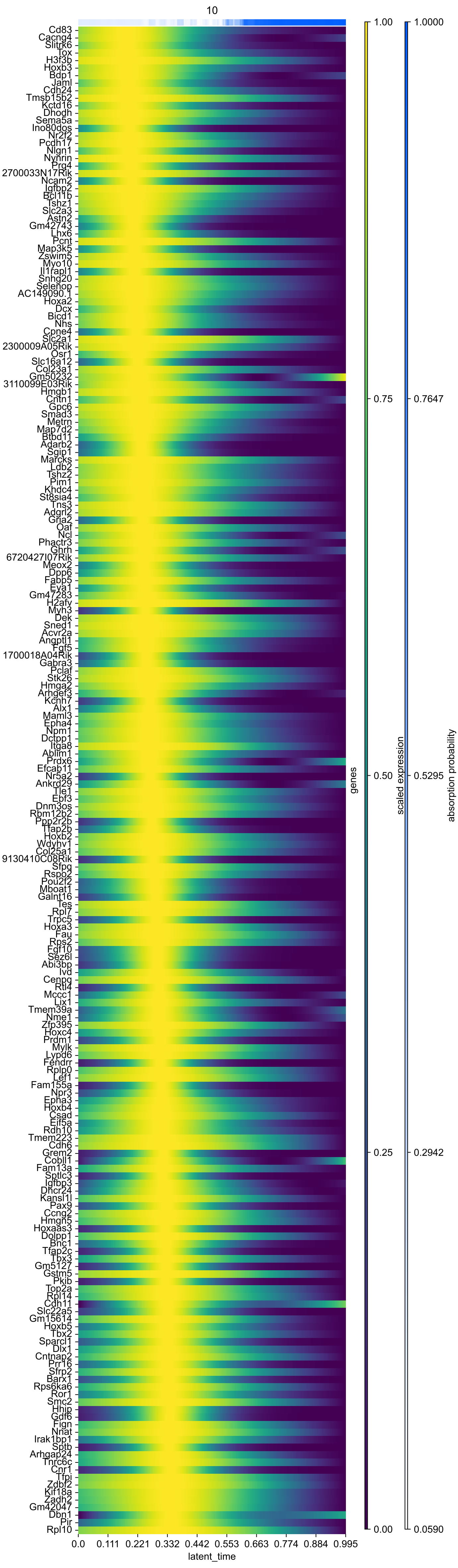

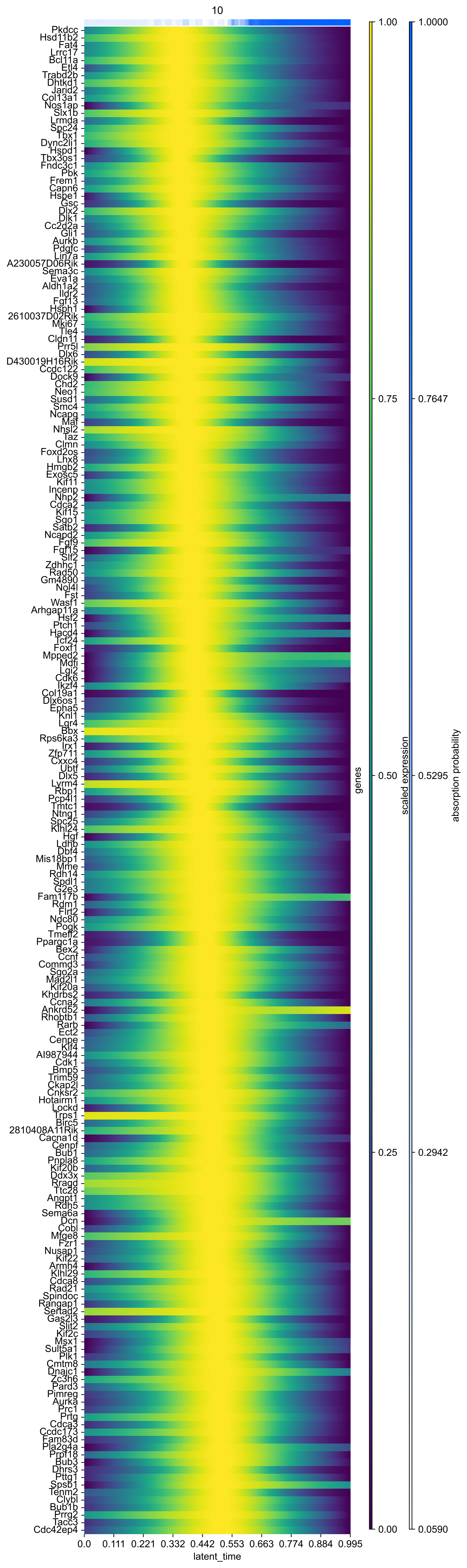

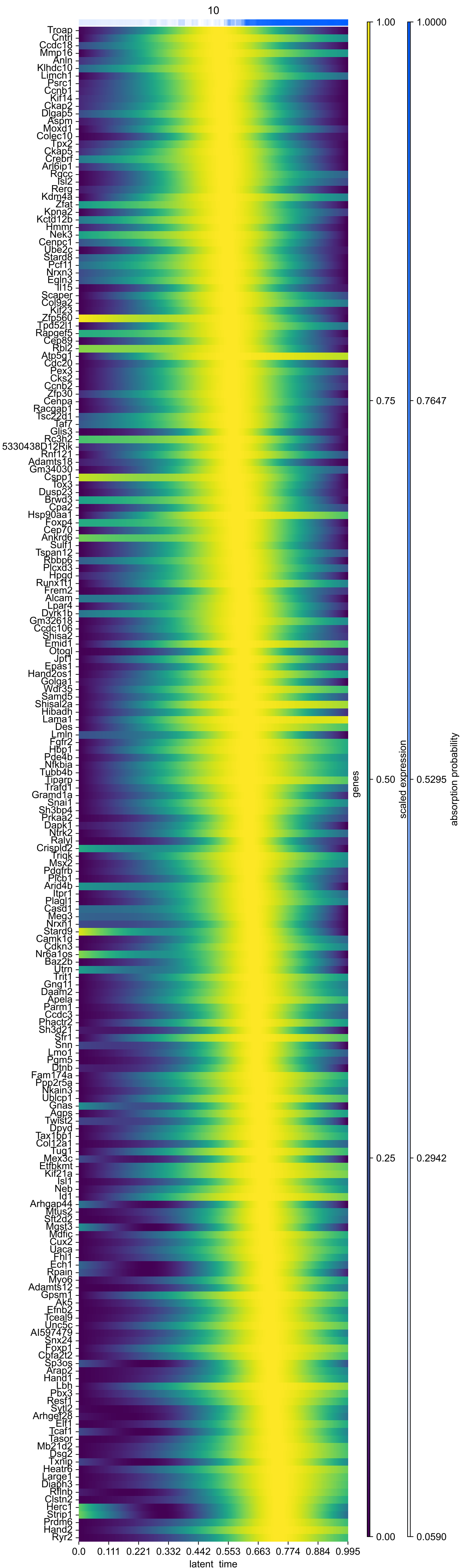

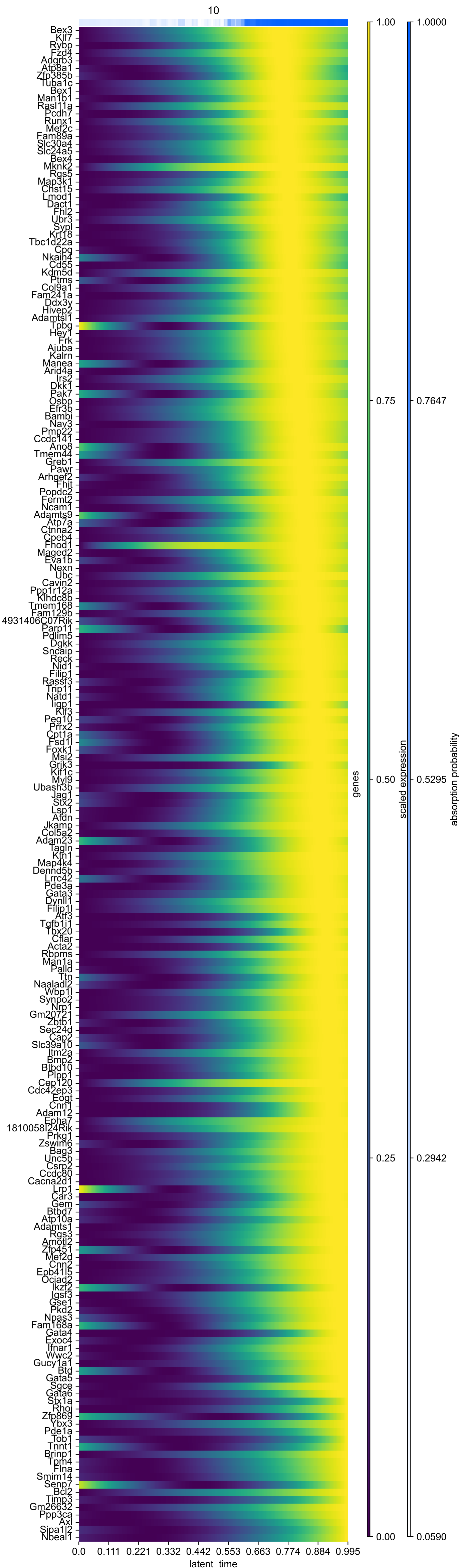

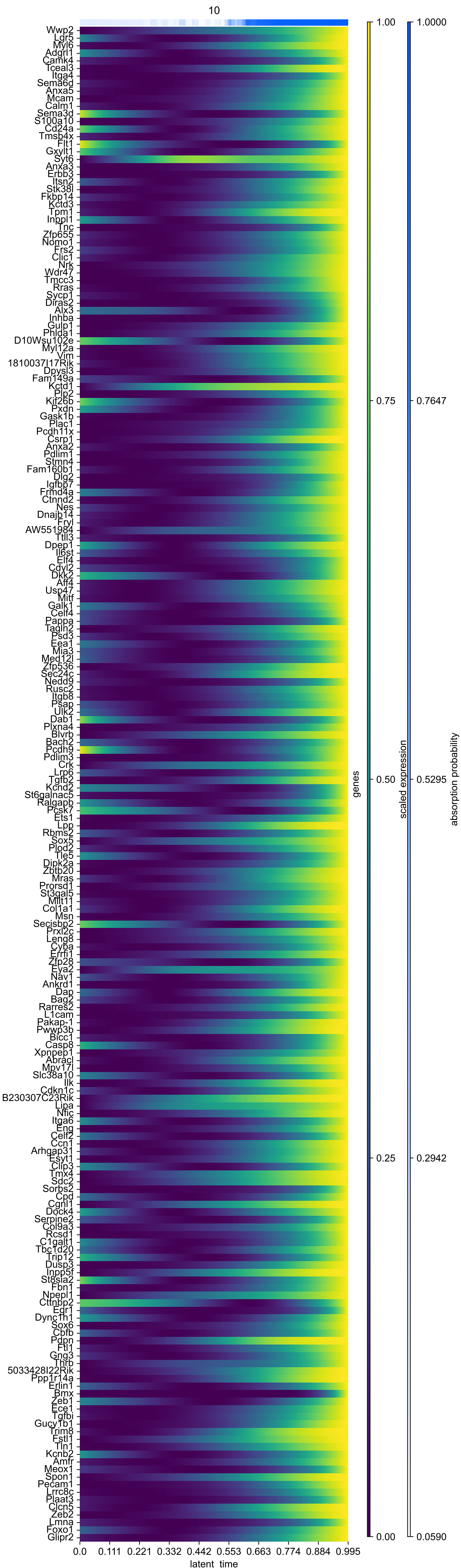

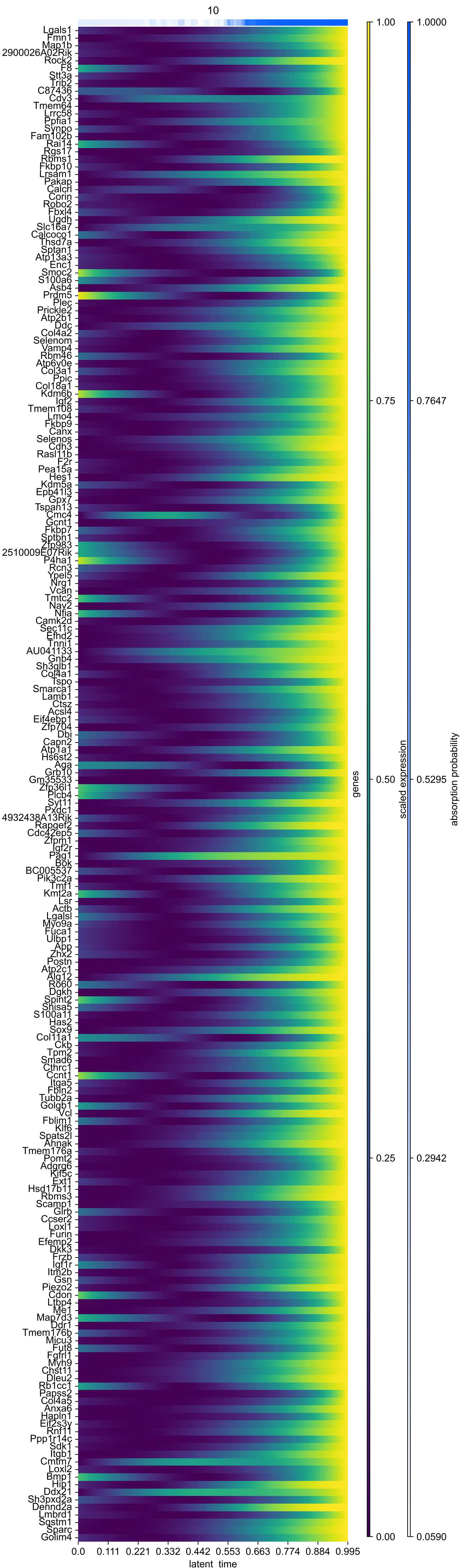

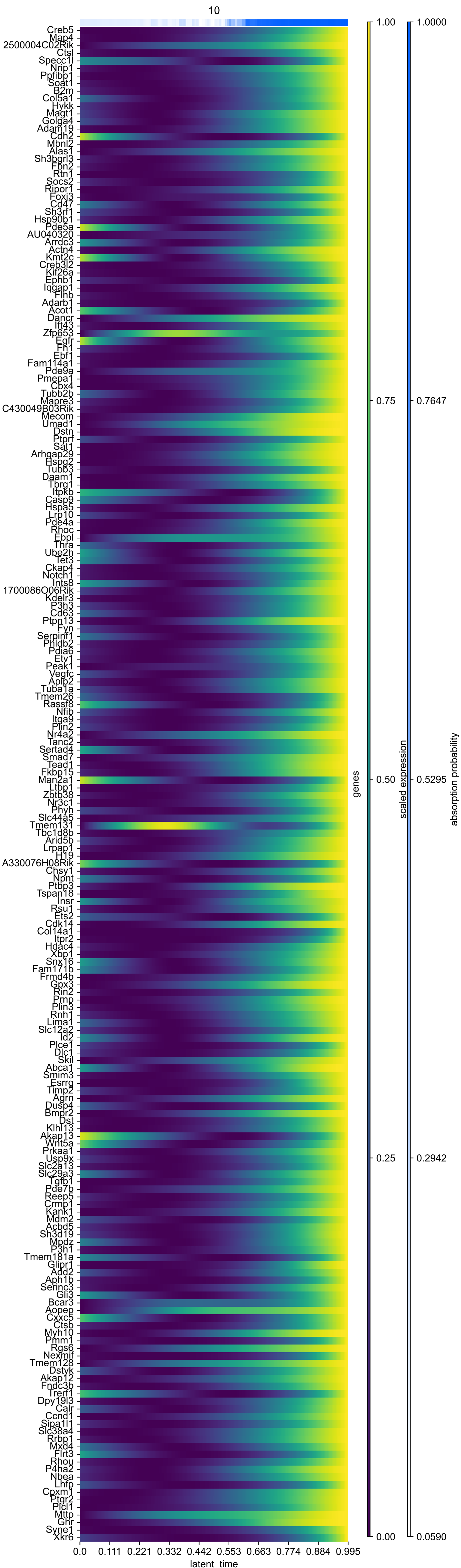

Supplement: Supplementary file 8 — Supplementary Data 5 [file 41467_2023_37015_MOESM8_ESM.pdf]
