# Supplementary material for: Single-cell transcriptomics uncovers a non-autonomous Tbx1-dependent genetic program controlling cardiac neural crest cell development
Source: Nat Commun. 2023 Mar 21;14:1551. doi: 10.1038/s41467-023-37015-9 (PMC10027855; doi:10.1038/s41467-023-37015-9)
Supplement: Supplementary file 15 — Reporting Summary [file 41467_2023_37015_MOESM15_ESM.pdf]

nature portfolio

Corresponding author(s): Bernice Morrow, Christopher De Bono

Last updated by author(s): Feb 10, 2023

Reporting Summary

Nature Portfolio wishes to improve the reproducibility of the work that we publish. This form provides structure for consistency and transparency in reporting. For further information on Nature Portfolio policies, see our Editorial Policies and the Editorial Policy Checklist.

Statistics

For all statistical analyses, confirm that the following items are present in the figure legend, table legend, main text, or Methods section.

n/a	Confirmed	
☐	☒	The exact sample size (*n*) for each experimental group/condition, given as a discrete number and unit of measurement
☐	☒	A statement on whether measurements were taken from distinct samples or whether the same sample was measured repeatedly
☐	☒	The statistical test(s) used AND whether they are one- or two-sided<br>*Only common tests should be described solely by name; describe more complex techniques in the Methods section.*
☐	☒	A description of all covariates tested
☐	☒	A description of any assumptions or corrections, such as tests of normality and adjustment for multiple comparisons
☒	☐	A full description of the statistical parameters including central tendency (e.g. means) or other basic estimates (e.g. regression coefficient) AND variation (e.g. standard deviation) or associated estimates of uncertainty (e.g. confidence intervals)
☐	☒	For null hypothesis testing, the test statistic (e.g. *F*, *t*, *r*) with confidence intervals, effect sizes, degrees of freedom and *P* value noted<br>*Give P values as exact values whenever suitable.*
☒	☐	For Bayesian analysis, information on the choice of priors and Markov chain Monte Carlo settings
☐	☒	For hierarchical and complex designs, identification of the appropriate level for tests and full reporting of outcomes
☒	☐	Estimates of effect sizes (e.g. Cohen's *d*, Pearson's *r*), indicating how they were calculated

Our web collection on statistics for biologists contains articles on many of the points above.

Software and code

Policy information about availability of computer code

Data collection

Immunofluorescence staining on paraffin sections:
Embryo sections were imaged using a Zeiss Axio Imager M2 microscope.

Immunofluorescence on wholemount embryos:
Embryos were imaged as a Z-stack using a Nikon CSU-W1 spinning confocal microscope.

RNAscope on wholemount embryos:
Embryos were imaged as a Z-stack using a Leica SP5 confocal microscope or a Nikon CSU-W1 spinning confocal microscope.

RNAscope on cryosections:
Section were imaged using a Zeiss Axio Imager M2 microscope.

Histological sections:
Sections were imaged with a Zeiss Axioskop 2 plus microscope.

Intracardiac India ink injections:
Images were taken using a Leica MZ125 stereomicroscope.

FACS:
A BD FACSAria II (BD Biosciences) system was used.

Next generation sequencing:
Sequencing of the DNA libraries was performed using an Illumina HiSeq 4000 system or NovaSeq 6000.

Data analysis

Data analysis:
Immunofluorescence staining on paraffin sections and RNAscope on cryosections:
Acquistion of images were analyzed using Zen software from Zeiss, ImageJ and Adobe Photoshop software.

Immunofluorescence on wholemount and RNAscope on wholemount embryos:
Acquistion of images were analyzed using ImarisViewer 9.8.0 and ImageJ software.

Histological sections and intracardiac ink injections:
Acquisition of images were analyzed using Zen software from Zeiss and Adobe Photoshop software.

FACS:
BD FACSDiva 8.0.1 software was used for flow cytometer data acquisition and analysis (https://www.bdbiosciences.com/en-us/products/software/instrument-software/bd-facsdiva-software).

scRNA-seq:
Cell Ranger v6.0.1, from 10x Genomics, was used to align scRNA-seq reads to the mouse reference genome (assembly and annotation, mm10-2020-A).
Seurat v4.0.5 was used to analyze data from individual scRNA-seq samples (Butler et al., PMID: 29608179).
RISC v1.6 was used for data integration (Liu et al., PMID: 33767393), https://github.com/bioinfoDZ/RISC.
CellRank v1.5.1 was used to infer differentiation trajectories (Lange et al., PMID: 35027767). The analysis used RNA velocity from Velocyto v0.17.17 and scVelo v0.2.4 (Bergen et al., PMID: 32747759).
CellChat v1.1.3 was used to identify the ligand-receptor interactions (Jin et al., PMID: 33597522).

For manuscripts utilizing custom algorithms or software that are central to the research but not yet described in published literature, software must be made available to editors and reviewers. We strongly encourage code deposition in a community repository (e.g. GitHub). See the Nature Portfolio guidelines for submitting code & software for further information.

Data

Policy information about availability of data

All manuscripts must include a data availability statement. This statement should provide the following information, where applicable:

- Accession codes, unique identifiers, or web links for publicly available datasets
- A description of any restrictions on data availability
- For clinical datasets or third party data, please ensure that the statement adheres to our policy

All scRNA-seq datasets generated in this study have been released. They are available under the accession number: GSE210521 with the link of https://www.ncbi.nlm.nih.gov/geo/query/acc.cgi?acc=GSE210521.
The reference genome mm10-2020-A was used.

Human research participants

Policy information about studies involving human research participants and Sex and Gender in Research.

Reporting on sex and gender	N/A
Population characteristics	N/A
Recruitment	N/A
Ethics oversight	N/A

Note that full information on the approval of the study protocol must also be provided in the manuscript.

Field-specific reporting

Please select the one below that is the best fit for your research. If you are not sure, read the appropriate sections before making your selection.

☒ Life sciences ☐ Behavioural & social sciences ☐ Ecological, evolutionary & environmental sciences

For a reference copy of the document with all sections, see nature.com/documents/nr-reporting-summary-flat.pdf

Life sciences study design

All studies must disclose on these points even when the disclosure is negative.

Sample size	For single-cell RNA-seq, sample sizes were estimated based upon requirements for optimal cell numbers. Sample sizes for scRNAseq were provided in Supplementary Table 1 of the manuscript. In situ hybridization, immunofluorescence and histology studies were done in n=3 or n>3 embryos. The exact number of embryos used for each experiment is indicated in the manuscript. No statistical methods were used to determine sample size, n equal or superior to 3 samples is the standard used by convention in the research field.
Data exclusions	Exclusion criteria were not pre-established. All data was included from the initial analyses and they are provided in GEO and Supplementary Data files.
Replication	Our replication attempts were successful. All RNAscope, histology and immunofluorescence experimental findings were reproduced independently 3 times or more. We performed 2 replicates of scRNA-seq of control and Tbx1 null embryos at E10.5. We performed one replicate of scRNA-seq of E8.5 and E9.5 in control (and Tbx1 null) embryos, but we compared with the literature of known marker genes to validate findings.
Randomization	Mice were randomly allocated into experimental groups based upon genotype. Female and male mice for genetic crosses were sorted randomly, at 2-6 months of age, during the peak of reproduction. Mouse embryos were genotyped to determine whether they were mutant vs wildtype. Further, we examined the embryos to make sure that genotype matched phenotype.
Blinding	We genotyped mice and embryos to know their affection status and this was needed to do functional genomic studies. Blinding was not possible during collection of control and mutant embryos because of visible phenotype differences between them. However, all subsequent analyses were unbiased. scRNA-seq uses software that provides unbiased clustering and peaks.

Reporting for specific materials, systems and methods

We require information from authors about some types of materials, experimental systems and methods used in many studies. Here, indicate whether each material, system or method listed is relevant to your study. If you are not sure if a list item applies to your research, read the appropriate section before selecting a response.

Materials & experimental systems

n/a	Involved in the study	
☐	☒	Antibodies
☒	☐	Eukaryotic cell lines
☒	☐	Palaeontology and archaeology
☐	☒	Animals and other organisms
☒	☐	Clinical data
☒	☐	Dual use research of concern

Methods

n/a	Involved in the study	
☒	☐	ChIP-seq
☐	☒	Flow cytometry
☒	☐	MRI-based neuroimaging

Antibodies

Antibodies used	The following primary antibodies were used: goat anti-GFP (1/200, Abcam ab6673), mouse anti-alpha smooth muscle actin (ACTA2) (1/200, Abcam ab7817), rabbit anti-αSMA (ACTA2; 1/200, Abcam ab5694), mouse anti-ISL1/2 (1/100, DSHB 40.2D6 and DSHB 39.4D5), mouse anti-TBX2 (1/100, Santa Cruz sc514291), rabbit anti-TAGLN (1/200, Abcam ab14106), rabbit anti-pSMAD1/5/9 (1/100, Cell Signaling D5B10 #13820), rabbit anti-TBX1 (1/100, Lifescience LS-C31179), rabbit anti-pERK (1/100, Cell Signaling 9101) and MF20 (1/100, Sarcomeric Myosin heavy chain antibody, mouse, DSHB). The following secondary antibodies from Invitrogen (Thermo Fisher Scientific) raised in donkey were used at 1/200: anti-goat IgG Alexa Fluor 488 (A11055), anti-mouse IgG Alexa Fluor 555 (A31570), anti-mouse IgG Alexa Fluor 647 (A3157), anti-rabbit IgG Alex Fluor 555 (A31572), anti-rabbit IgG Alex Fluor 568 (A10042) and anti-rabbit IgG Alex Fluor 647 (A31573).
Validation	These are all validated antibodies in published articles.<br>- anti-GFP (Abcam ab6673): Fujimoto et al., 2010, PMID: 20963824; Shi et al., 2022, PMID: 34686881<br>- anti-αSMA (Abcam ab7817): Dai et al., 2013, PMID: 24131868<br>- anti-αSMA(Abcam ab5694): Shi et al., 2022, PMID: 34686881<br>- anti-ISL1/2 (DSHB 40.2D6 and DSHB 39.4D5): Mesbah et al., 2012, PMID: 22116936; Stefanovic et al., 2020, PMID: 32804075<br>- anti-TBX2 (Santa Cruz sc514291): Wojahn et al., 2019, PMID: 31870435<br>- anti-TAGLN (Abcam ab14106): Tang et al., 2020, PMID: 31883835<br>- anti-pSMAD1/5/9 (Cell Signaling D5B10): Hayano et al., 2015, PMID: 25742798; Skauli et al., 2022, PMID: 35518645<br>- anti-TBX1 (Lifescience LS-C31179): De Bono et al., 2018, PMID: 30016433<br>- anti-pERK (Cell Signaling 9101): Lu et al., 2022, PMID: 35831318<br>- MF20 (DSHB): Rana et al., 2014, PMID: 25190705 ; Stefanovic et al., 2020, PMID: 32804075<br>We also examined the literature for expression patterns that were expected of the antibodies.<br>ISL1, TBX2 and GFP immunostaining results were validated by RNAscope mRNA analysis in our manuscript.

Animals and other research organisms

Policy information about studies involving animals; ARRIVE guidelines recommended for reporting animal research, and Sex and Gender in Research

Laboratory animals	Wnt1-Cre, ROSA26R-GFPf/+, Tbx1+/- and Tbx1f/+ mice were maintained on a Swiss Webster genetic background. Tbx2f/+ and Tbx3f/+ mice were maintained on a mixed Swiss Webster and C57BL/6 genetic background. Adult breeding mice in our colony range 2-6 months of age. We did not genotype for sex of the embryos; embryos were used randomly depending on genotype. Mice were housed at a temperature between 20 and 22°C with a level of humidity between 40 and 60% and with a 14-hours light/ 10-hours dark cycle.
Wild animals	No wild animals were used.
Reporting on sex	We did not genotype for sex in the embryos because we are examining stages prior to sexual development.
Field-collected samples	No field collected samples were performed.
Ethics oversight	All experiments using mice were carried out according to regulatory standards defined by the National Institutes of Health and the Institute for Animal Studies, Albert Einstein College of Medicine (https://www.einsteinmed.org/administration/animal-studies/), IACUC protocol is #0000-1034.

Note that full information on the approval of the study protocol must also be provided in the manuscript.

Flow Cytometry

Plots

Confirm that:

☒ The axis labels state the marker and fluorochrome used (e.g. CD4-FITC).

☒ The axis scales are clearly visible. Include numbers along axes only for bottom left plot of group (a 'group' is an analysis of identical markers).

☒ All plots are contour plots with outliers or pseudocolor plots.

☒ A numerical value for number of cells or percentage (with statistics) is provided.

Methodology

Sample preparation	Embryos were collected and microdissected in 1x PBS at 4°C. Dissected tissues of interest were maintained in DMEM (Dulbecco's Modified Eagle Medium, GIBCO 11885084) at 4°C. For E8.5 embryos, the rostral half of the embryos including the heart was collected. At E9.5, the pharyngeal region from pharyngeal arches 1 onwards plus the heart were collected. The head of the embryos were removed at E9.5. At E10.5, the pharyngeal region plus heart were collected. The head and pharyngeal aches 1 were removed at E10.5. Then, tissues were incubated in 1ml of 0.25% Trypsin-EDTA (GIBCO, 25200056) containing 50U/ml of DNase I (Millipore, 260913-10MU), at room temperature for 7 minutes. Next, heat inactivated FBS (Fetal Bovine Serum, ATCC, 30-2021) was added to stop the reaction at a final concentration of 10%, at 4°C. Dissociated cells were centrifugated for 5 minutes at 300 x g at 4°C and the supernatant was removed. Cells were then resuspended in 1x PBS without Ca2+ and Mg2+ (Coming, 21-031-cv) containing 10% FBS at 4°C and filtered with a 100µm cell strainer. A total of 1µl DAPI (1mM) (Thermo Fisher Scientific, D3571) was added before FACS.
Instrument	Cells were FACS purified and collected using a BD FACSAria II (BD Biosciences) system.
Software	BD FACSDiva 8.0.1 software (BD Biosciences) was used.
Cell population abundance	Total number of FACS purified cells are provided in Supplementary Figure 15. For all samples, high purity of GFP positive neural crest cells is validated by detection of specific genes expressed in neural crest cells in our scRNA-seq data analysis.
Gating strategy	GFP positive and DAPI negative cells corresponding to viable neural crest cells were sorted. The gating strategy is presented in Supplementary Figure 15. We show FSC-A/SSC-A (FSC-A, forward scatter area; SSC-A, side scatter area) plot with gated as cells. Singlets were gated in FSC-A/FSC-H (FSC-area, FSC-height) plot and in SSC-A/SSC-H (SSC-area; SSC-height) plot. The viable cells were gated in FSC-A/Pacific Blue-A plot. GFP positive cells were gated in GFP-A/SSC-A plot.

☒ Tick this box to confirm that a figure exemplifying the gating strategy is provided in the Supplementary Information.
